# Resolving the APN controversy in PEDV infection: Comparative kinetic characterization through single-virus tracking

**DOI:** 10.1371/journal.ppat.1013317

**Published:** 2025-06-30

**Authors:** Da An, Yan Li, Yi Pei, Yong-Hao Ma, Fei Yao, Yongzhi Feng, Yanke Shan, Fei Liu

**Affiliations:** 1 Joint International Research Laboratory of Animal Health and Food Safety of Ministry of Education, Single Molecule Biochemistry & Biomedicine Laboratory (Sinmolab), Nanjing Agricultural University, Nanjing, Jiangsu, China; 2 Sanya Institute of Nanjing Agricultural University, Sanya, Hainan, China; Loyola University Chicago Stritch School of Medicine, UNITED STATES OF AMERICA

## Abstract

Aminopeptidase N (APN) plays multiple roles in various physiological processes, with its function as a viral receptor in several coronaviruses being one of the most prominent. However, the role of porcine APN (pAPN) in porcine epidemic diarrhea virus (PEDV) has remained controversial. Single-virus tracking enables a more comprehensive dynamic dissection of pAPN utilization during virus entry. In this study, a comparative analysis of pAPN usage by PEDV, transmissible gastroenteritis virus (TGEV), and swine acute diarrhea syndrome coronavirus (SADS-CoV) provides more precise and quantitative insights into pAPN’s specific role in PEDV entry. Here, we used molecular docking and surface plasmon resonance (SPR) to demonstrate that pAPN binds to PEDV, with lower affinity than to TGEV. However, pAPN facilitates PEDV replication through internalization only in susceptible cells, not in non-susceptible cells. Using single-virus tracking, we observed that pAPN triggers PEDV internalization *via* clathrin- and caveolae-mediated endocytosis, resembling a receptor-mediated process. pAPN participates in 35% of PEDV internalization events, but mediates 80% of TGEV internalization, with pAPN-mediated PEDV internalization occurring approximately 60 s slower than TGEV. The dynamic differences in the internalization of PEDV and TGEV mediated by pAPN primarily arise during the binding stage prior to the initiation of accelerated directional movement, whereas their durations of movement are comparable. Additionally, we found that the internalization dynamics of porcine deltacoronavirus (PDCoV), which also uses pAPN as a receptor, are similar to those of TGEV. These findings resolve the controversy surrounding pAPN’s role in PEDV entry, and highlight the dynamic differences in PEDV, TGEV, PDCoV, and SADS-CoV internalization *via* pAPN at single-virus level, providing a novel theoretical basis for the potential receptor evaluation from kinetic perspective, which could significantly contribute to the development of strategies against future PEDV outbreaks.

## Author summary

Porcine epidemic diarrhea virus (PEDV), a highly contagious coronavirus, continues to inflict substantial economic losses on the global swine industry. While aminopeptidase N (APN) is well-established as a functional receptor for several coronaviruses, the involvement of porcine APN (pAPN) in PEDV infection has remained controversial. Through comparative analysis of pAPN usage by PEDV, transmissible gastroenteritis virus (TGEV), and swine acute diarrhea syndrome coronavirus (SADS-CoV), we have conclusively demonstrated that pAPN, while not serving as a receptor for PEDV, significantly facilitates viral replication in susceptible cells through enhanced internalization mechanisms. Utilizing single-virus tracking, we revealed that pAPN mediates PEDV internalization *via* clathrin- and caveolae-mediated endocytosis, resembling a receptor-mediated process, albeit with markedly lower efficiency compared to its interaction with TGEV. Additionally, we found that the internalization dynamics of PDCoV mediated by pAPN are similar to those of TGEV. These findings not only resolve the controversy surrounding pAPN’s role in PEDV entry but also establish a kinetic framework for potential receptor evaluation, thereby providing crucial molecular insights for the development of targeted antiviral interventions against PEDV infection.

## Introduction

Aminopeptidase N (APN), a type II transmembrane glycoprotein, plays multifunctional roles in physiological processes, particularly in virus infections, including serving as a primary receptor to mediate virus entry, processing peptides to modulate immune signaling, and influencing host susceptibility to virus infections [1]. APN homologs have been identified as receptors for several alphacoronaviruses, such as transmissible gastroenteritis virus (TGEV) [2], human coronaviruses 229E (HCoV-229E) [3], feline enteric coronavirus (FCoV), and canine enteric coronavirus (CCoV) [4], as well as for porcine deltacoronavirus (PDCoV) [5]. Notably, a recent study demonstrated that APNs from eight out of seventeen animal species, including dogs, can bind to the receptor-binding domains (RBDs) of PDCoV and TGEV [6], highlighting the critical role of APN in the potential cross-species transmission of coronaviruses. Porcine epidemic diarrhea virus (PEDV), which also belongs to alphacoronaviruses, has a spike (S) protein structure similar to that of other coronaviruses (CoVs) known to use APN as a receptor [7]. However, whether pAPN serves as a receptor for PEDV remains controversial.

PEDV is considered the most harmful swine enteric coronavirus, causing severe gastrointestinal disease with a mortality rate of 50% - 90% in piglets. This has led to significant economic losses in the swine industry [8]. Therefore, research on the infection mechanisms of PEDV is essential to provide insights into the pathogenesis, evolution, and transmission. The initial and essential step of viral infection is the binding of the S protein to host receptors on the surface. Previous studies have suggested that PEDV might utilize porcine APN (pAPN) as a receptor, as anti-pAPN antibodies can disrupt PEDV infection [9]. Moreover, the overexpression of exogenous pAPN in both cells and pAPN transgenic mice increased susceptibility to PEDV infection [10]. However, more recent research has produced conflicting results. Swine testis (ST) and HeLa cells with endogenous pAPN showed little to no sensitivity to PEDV infection [11,12], and notably, PEDV can infect pAPN-negative African green monkey kidney epithelial (Vero) cells [13]. Furthermore, APN-knockout pigs did not show resistance to PEDV infection [14]. Therefore, the exact role of pAPN in PEDV infection remains unclear.

The controversy surrounding the role of pAPN in PEDV may arise from limitations of the traditional analytical methods that typically offer static and endpoint results, which are incapable of capturing the dynamic molecular details of underlying processes [15]. Single-virus tracking enables in situ, real-time observation of individual viral particles as they enter live cells, offering direct evidence of virus-host interactions at the subcellular level [16,17]. Its remarkable ability to elucidate dynamic invasion processes has been demonstrated in previous studies on the entry of severe acute respiratory syndrome coronavirus 2 (SARS-CoV-2) mediated by angiotensin-converting enzyme 2 (ACE2) [18] and influenza virus mediated by sialic acid receptors [19].

In this study, we employed single-virus tracking to quantitatively investigate the exact role of pAPN in PEDV infection. Given that TGEV uses pAPN as a receptor, while swine acute diarrhea syndrome coronavirus (SADS-CoV) does not utilize pAPN for infection [20], we conducted a comparative analysis of PEDV, TGEV, and SADS-CoV. Molecular docking and surface plasmon resonance (SPR) showed that pAPN has binding affinity for the PEDV spike protein. We found that of pAPN promotes PEDV infection by mediating internalization in susceptible cells, but not in nonsusceptible cells. Single-virus tracking revealed that pAPN mediates PEDV internalization by initiating clathrin-mediated endocytosis (CME) and caveolae-mediated endocytosis (CavME), similar to its role in TGEV internalization. However, pAPN initiates PEDV internalization less efficiently than TGEV, participating in only 35% of PEDV internalization, compared to 80% for TGEV. pAPN-mediated PEDV internalization occurs approximately 60 seconds (s) slower than TGEV, and we found that the dynamic differences between pAPN-mediated PEDV and TGEV internalization are mainly due to the binding stage before initiating accelerated directional movement, whereas their durations of movement are comparable. Additionally, we also observed that the internalization dynamics of PDCoV, which also uses pAPN as a receptor, are similar to those of TGEV. In summary, our study resolves the controversy surrounding pAPN’s role in PEDV entry *via* single-virus tracking, and highlights the distinct dynamic characteristics of pAPN in the internalization of PEDV, TGEV, PDCoV and SADS-CoV, providing a theoretical basis for receptor evaluation from kinetics perspective.

## Results

### Binding affinity of PEDV S protein for pAPN revealed by docking structure and SPR

**Fig 1:**
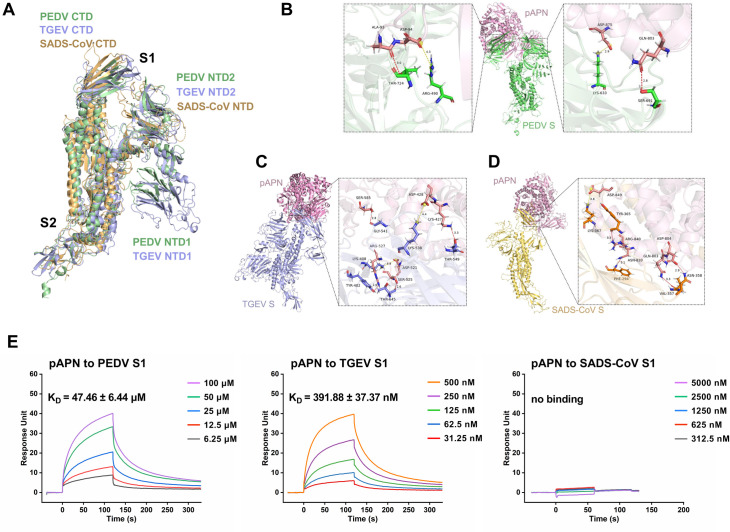
Binding affinity of pAPN with the S proteins of PEDV, TGEV, and SADS-CoV revealed by docking structure and SPR. (A) Structural alignment of the S proteins from PEDV, TGEV and SADS-CoV. (B) Docking structure and atomic details illustrating the interaction between PEDV S protein (green) and pAPN (pink). (C) Docking structure and atomic details illustrating the interaction between TGEV S protein (purple) and pAPN (pink). (D) Docking structure and atomic details illustrating the interaction between SADS-CoV S protein (yellow) and pAPN (pink). Contacting residues are represented as sticks, with hydrogen bonds shown as red lines and salt bridges as yellow lines. (E) Binding affinities of pAPN to the S1 proteins of PEDV, TGEV, and SADS-CoV measured by SPR.

The three-dimensional structure of the CoV S protein plays a key role in receptor recognition [21]. In this study, we first performed a structural alignment of the PEDV S protein with those of TGEV and SADS-CoV. Since the structure of the TGEV S protein remains unreported, we used the I-TASSER platform to predict its three-dimensional structure before alignment. Alignment results showed that the C-terminal domain (CTD) of the S1 proteins of PEDV and TGEV contain two layers of β-sheets, whereas the CTD of SADS-CoV S1 protein only has one twisted β-sheet (Fig 1A). Due to gene duplication, both the S1 proteins of PEDV and TGEV contain a second N-terminal domain (NTD), while SADS-CoV S1 protein contains only one NTD. In contrast to the NTD and CTD of S1 protein having unique structural features, the S2 proteins of the three viruses were structurally conserved and aligned well. Moreover, we further aligned the amino acid sequences of the S protein from the three viruses and performed a homology analysis (S1A Fig). The results showed that the amino acid homology between the S proteins of PEDV and TGEV was 31.5%, while the homology between the S proteins of PEDV and SADS-CoV was only 18.8% (S1B Fig). Therefore, these results indicate that the structural and sequence basis of the PEDV S protein is more similar to that of TGEV than to SADS-CoV.

To investigate the binding of pAPN with PEDV S protein, we first conducted the molecular docking analysis of pAPN with the S proteins of PEDV, TGEV, and SADS-CoV, respectively. Hydrogen bonds and salt bridges between key contact residues were discovered in the docking structures of pAPN with the S proteins of three viruses. Within a distance cutoff of 5 Å, the docking structure analysis identified four residues (T724, R490, K633 and S691) in the PEDV S protein that interact with pAPN (Fig 1B), while six residues (G541, K408, Y402, T645, K538 and T549) in the TGEV S protein were found to interact with pAPN (Fig 1C). Similarly, residues interacting through hydrogen bonds or salt bridges (K367, Y365, F294, V357, N358) also exhibited in the docking structure between pAPN and SADS-CoV S protein (Fig 1D). We further predicted the binding affinity between pAPN and the S proteins of the three viruses using the Rosetta Interface-Analyzer. Typically, docking structures of proteins with a free binding energy below -15 kcal/mol are considered to exhibit a binding propensity [22]. It showed that the free binding energies of pAPN with the S proteins of PEDV, TGEV and SADS-CoV were -32.406, -48.748, and -7.892 kcal/mol, respectively (S1C Fig). Additionally, the binding affinity between pAPN and PDCoV S protein was used to validate the accuracy of molecular docking (S2 Fig), with a free binding energy is -37.840 kcal/mol, indicating that pAPN exhibits a strong binding affinity for PDCoV, consistent with previous reports. Lower free binding energy indicating more stable binding and stronger affinity, although the free binding energy of PEDV with pAPN (-32.406 kcal/mol) is higher than those of TGEV (-48.748 kcal/mol) and PDCoV (-37.840 kcal/mol), PEDV shows a binding propensity for pAPN, especially in comparison to SADS-CoV (-7.892 kcal/mol). Although molecular docking provides valuable structural insights and preliminary predictions of potential interactions, it does not allow for quantitative assessment of binding strength. We then performed surface plasmon resonance (SPR) to directly evaluate the binding affinities between pAPN and the S1 proteins of PEDV, TGEV, and SADS-CoV. The SPR results showed that pAPN binds to PEDV S1 protein with a lower affinity (KD = 47.46 ± 6.44 μM) compared to TGEV S1 protein (K_D_ = 391.88 ± 37.37 nM), while no detectable binding was observed between pAPN and SADS-CoV S1 protein (Fig 1E). The binding energy differences between pAPN-PEDV and pAPN-TGEV, derived from the dissociation constants obtained by SPR, were in good agreement with those predicted by molecular docking analyses. This consistency supports the validity of the proposed interaction models of molecular docking. The binding affinity between PEDV and pAPN suggests that pAPN may play an important role in PEDV infection, despite not being the primary receptor for the virus.

### pAPN promotes PEDV replication in susceptible but not nonsusceptible cells

**Fig 2:**
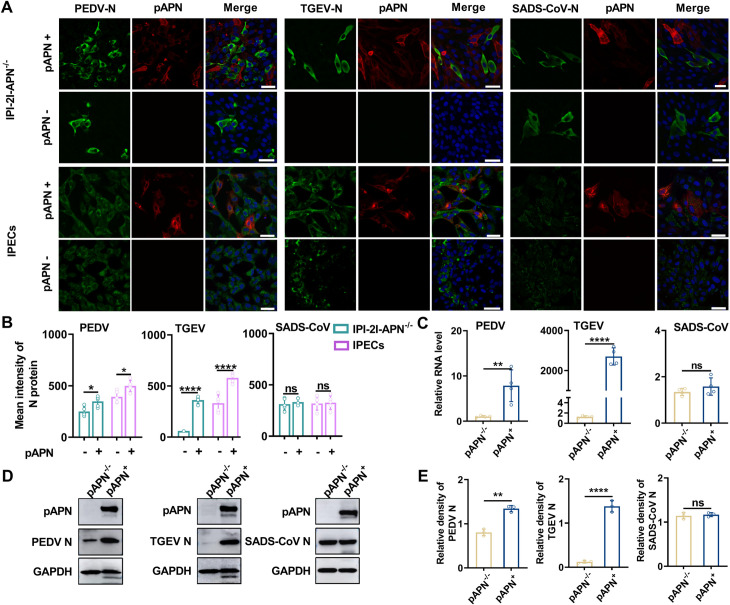
The role of pAPN in the infection of PEDV, TGEV and SADS-CoV. (A) Effect of pAPN on PEDV, TGEV, and SADS-CoV infection in IPI-2I-APN^-/-^ cells and IPECs. Cells were infected with PEDV, TGEV and SADS-CoV (MOI = 0.1) in the presence of 5 μg/mL trypsin. The N proteins of PEDV, TGEV and SADS-CoV were stained using IFA at 24 hpi. Scale bar, 50 μm. (B) Mean intensity of the N protein from (A) was quantified (n = 5 cells; mean ± SD). (C) IPI-2I-APN^-/-^ cells re-expressing pAPN were infected with PEDV, TGEV and SADS-CoV (MOI = 0.1), and relative viral RNA levels were quantified by RT-qPCR at 24 hpi (n = 3 independent experiments; mean ± SD). (D) Western blotting of N proteins in IPI-2I-APN^-/-^ cells re-expressing pAPN after infection with PEDV, TGEV and SADS-CoV (MOI = 0.1) at 24 hpi. (E) Relative density of the N proteins from (D) (n = 3 independent experiments; mean ± SD). Non-transfected infected cells served as controls in all independent experiments. Two-tailed *P*-values were calculated by unpaired Student’s t test. *P < *0.05 was considered significant (ns *P *≥ 0.05, *P < 0.05, **P < 0.01, ***P < 0.001, ****P < 0.0001).

Next, we investigated whether the binding affinity between PEDV and pAPN, revealed by molecular docking and SPR, ultimately promotes PEDV infection in various cells. Here, we conducted an immunofluorescence assay (IFA) to visualize the effect of pAPN on PEDV, TGEV, and SADS-CoV infection in IPI-2I-APN^-/-^ cells lacking endogenous APN and intestinal porcine epithelial cells (IPECs) expressing endogenous APN. Fluorescence images showed that in IPI-2I-APN^-/-^ cells, PEDV infection was reduced but not abolished, whereas TGEV infection was completely inhibited. Notably, re-expression of pAPN in the knockout cells significantly enhanced PEDV replication and fully restored TGEV infection. Moreover, pAPN also significantly promoted the infection of PEDV and TGEV in IPECs, suggesting that pAPN promotes PEDV replication in susceptible cells, even in IPI-2I-APN^-/-^ cells lacking endogenous pAPN (Fig 2A and 2B). In addition, pAPN had no effect on SADS-CoV infection in either IPECs or IPI-2I-APN^-/-^ cells, consistent with SADS-CoV not utilizing pAPN as a receptor for entry (Fig 2A and 2B). We then performed RT-qPCR and western blotting assays in IPI-2I-APN^-/-^ cells at 24 hours post-infection (hpi). RT-qPCR results showed that pAPN enhanced PEDV RNA levels by approximately 7.8-fold. In contrast, TGEV replication in IPI-2I-APN^-/-^ cells was exhibited a profound dependence on pAPN, with re-expression of pAPN resulting in a more than 2000-fold increase in RNA levels relative to cells lacking pAPN. Consistent with the IFA results, pAPN had no effect on SADS-CoV infection (Fig 2C). Western blotting analysis further confirmed that pAPN significantly promoted PEDV replication in IPI-2I-APN^-/-^ cells, despite PEDV being able to infect cells even without pAPN (Fig 2D and 2E). Moreover, pAPN rescued TGEV infection in IPI-2I-APN^-/-^ cells, while the presence or absence of pAPN had no effect on the susceptibility to SADS-CoV. Importantly, we further evaluated the role of pAPN in PEDV infection in Caco2 cells, and the results showed that PEDV failed to infect these cells, even with pAPN overexpression (S3A Fig). However, pAPN overexpression allowed TGEV to infect Caco2 cells (S3B Fig). Overall, these findings suggest that pAPN facilitates PEDV replication in susceptible cells but could not rescue PEDV infection in non-susceptible cells.

### pAPN facilitates PEDV infection *via* internalization not attachment

**Fig 3:**
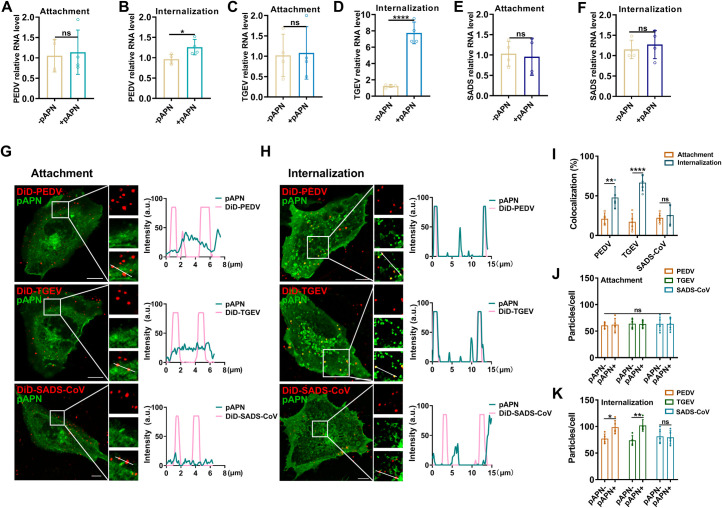
pAPN promotes PEDV and TGEV internalization rather than attachment. (A-F) The roles of pAPN in the attachment (0 mpi) and internalization (60 mpi) of PEDV, TGEV, and SADS-CoV were assessed using RT-qPCR (n = 4 experiments; mean ± SD). Control represents the viral RNA levels in cells without pAPN transfection. (G and H) Colocalization fluorescence imaging of DiD-PEDV, DiD-TGEV and DiD-SADS-CoV with pAPN during the attachment and internalization stages. Scale bar, 10 µm. (I) Colocalization analysis of DiD-PEDV, DiD-TGEV and DiD-SADS-CoV with pAPN in (G and H) (three experiments; n = 5 cells; mean ± SD). (J and K) The number of particles of the three viruses per cell during the attachment and internalization stages. Two-tailed *P*-values were calculated using an unpaired Student’s t test. *P* < 0.05 was considered significant (ns *P* ≥ 0.05, *P < 0.05, **P < 0.01, ***P < 0.001, ****P < 0.0001).

To investigate whether pAPN mediates PEDV infection by promoting viral entry, we conducted comparative experiments involving PEDV, TGEV, and SADS-CoV. IPECs with or without pAPN overexpression were infected with a 10 multiplicity of infection (MOI) of each virus at 4 °C for 30 min to allow attachment, followed by incubation at 37 °C for 1 h to internalize. The RT-qPCR results showed that pAPN overexpression did not significantly affect the attachment of PEDV, TGEV or SADS-CoV (Fig 3A, 3C and 3E). However, pAPN overexpression significantly enhanced PEDV internalization by 1.2-fold (Fig 3B) and TGEV internalization by 7-fold (Fig 3D) compared to the controls, whereas no significant effect was observed on SADS-CoV internalization (Fig 3F). These findings emphasize that pAPN promotes PEDV and TGEV infection through internalization rather than attachment. To visualize pAPN-mediated virus internalization, we labeled the lipid envelopes of PEDV, TGEV, SADS-CoV, and PDCoV with the lipophilic dye 1,1’-Dioctadecyl-3,3,3’,3’-tetramethylindodicarbocyanine (DiD). IFA confirmed that the virions were successfully labeled with DiD, with no effect on infectivity [15,22], indicating that this method effectively visualizes virions (S4 Fig). We then used confocal microscopy to visualize the colocalization of DiD-PEDV, DiD-TGEV and DiD-SADS-CoV with pAPN during the attachment and internalization stages. Fluorescence images showed low colocalization between pAPN and the three viruses during the attachment stage (Fig 3G). At 60 min post-infection (mpi), DiD-PEDV, DiD-TGEV and DiD-SADS-CoV were successfully internalized, and the fluorescence intensity profiles showed the obvious colocalization of DiD-PEDV and DiD-TGEV with pAPN, while DiD-SADS-CoV exhibited low colocalization with pAPN (Fig 3H). Statistical analysis revealed that the colocalization ratio of pAPN with PEDV and TGEV was significantly higher during the internalization stage than during the attachment stage, whereas SADS-CoV exhibited low colocalization with pAPN at both stages (Fig 3I). Moreover, pAPN significantly increased the number of PEDV and TGEV particles during the internalization stage, but not during the attachment stage. In contrast, pAPN did not affect the number of SADS-CoV particles at either the attachment or internalization stage (Fig 3J and 3K). These results reveal the unique role of pAPN in promoting the internalization rather than the attachment of PEDV, which is consistent with TGEV, suggesting similarity in pAPN-mediated entry among these viruses.

### pAPN initiates PEDV internalization pathways resembling a receptor

**Fig 4:**
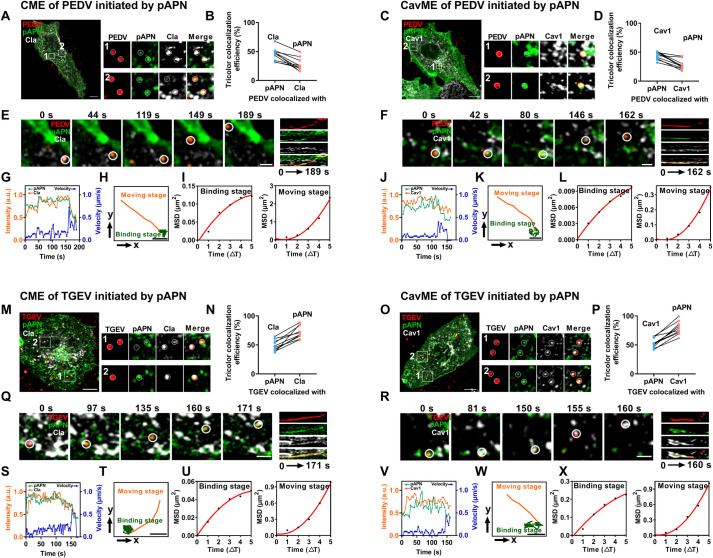
pAPN mediates PEDV and TGEV internalization *via* CME and CavME. (A-D) Colocalization images and triple-color colocalization ratio of PEDV, pAPN and Cla/Cav1 at 60 mpi. Scale bar, 10 µm. (E and F) Time-lapse images and kymographs (DiD-PEDV: red; pAPN: green; Cla/Cav1: white) showing PEDV internalization *via* CME and CavME initiated by pAPN. Scale bar, 2 µm. (G) Fluorescence intensities (green and orange lines) and velocities (blue line) of the circled PEDV in (E). (H) Trajectories of the circled PEDV in (E), showing the binding stage (green) and moving stage (orange). Scale bar, 2 µm. (I) MSD plots of the circled PEDV during the binding stage and moving stage in (E). (J) Fluorescence intensities (green and orange lines) and velocities (blue line) of the circled PEDV in (F). (K) Trajectories of the circled PEDV in (F), showing the binding stage (green) and moving stage (orange). Scale bar, 2 µm. (L) MSD plots of the circled PEDV during the binding stage and moving stage in (F). (M-P) Colocalization images and triple-color colocalization ratio of TGEV, pAPN and Cla/Cav1 at 60 mpi. Scale bar, 10 µm. (Q and R) Time-lapse images and kymographs (DiD-TGEV: red; pAPN: green; Cla/Cav1: white) showing TGEV internalization *via* CME and CavME initiated by pAPN. Scale bar, 2 µm. (S) Fluorescence intensities (green and orange lines) and velocities (blue line) of the circled TGEV in (Q). (T) Trajectories of the circled TGEV in (Q), showing the binding stage (green) and moving stage (orange). Scale bar, 2 µm. (U) MSD plots of the circled TGEV during the binding stage and moving stage in (Q). (V) Fluorescence intensities (green and orange lines) and velocities (blue line) of the circled TGEV in (R). (W) Trajectories of the circled TGEV in (R), showing the binding stage (green) and moving stage (orange). Scale bar, 2 µm. (X) MSD plots of the circled TGEV during the binding stage and moving stage in (R).

Activation of endocytic signaling is caused by receptor binding, and both CME and CavME are extensively used by enveloped viruses for internalization. Previous studies have demonstrated that PEDV, TGEV, and SADS-CoV enter cells through CME and CavME [16,23,24]. To determine whether pAPN is involved in initiating CME and CavME of PEDV, we evaluated the colocalization of DiD-PEDV with pAPN and clathrin (Cla)/caveolin-1 (Cav1) in IPECs at 60 mpi. Fluorescence images showed that DiD-PEDV colocalized with pAPN, as well as with clathrin-coated pits (CCPs) or caveolae, which was confirmed by triple-color colocalization analysis (Fig 4A-4D). We then used single-virus tracking to uncover the dynamics of CME and CavME initiated by PEDV upon binding to pAPN. Representative time-lapse images and kymographs (Fig 4E and 4F, S1 Movie) showed that DiD-PEDV first binds pAPN at the attachment sites, followed by the recruitment of Cla/Cav1, leading to the *de novo* formation of CCPs/caveolae. Subsequently, DiD-PEDV was rapidly endocytosed, as revealed by the fluorescence intensities and trajectories (Fig 4G, 4H, 4J and 4K). pAPN-mediated PEDV internalization exhibited two stages: a binding stage with restricted movement and a moving stage with directed movement (Fig 4I and 4L). Similarly, we observed that TGEV also colocalized with pAPN and Cla/Cav1 during internalization (Fig 4M–4P), and we then visualized the dynamics of pAPN in the CME and CavME of TGEV *via* single-virus tracking. As shown in the time-lapse images and kymographs (Fig 4Q and 4R, S2 Movie), pAPN binding to TGEV activated the *de novo* formation of CCPs/caveolae for rapid internalization, consistent with the results observed for PEDV. DiD-TGEV colocalized with pAPN and CCPs/caveolae at the viral binding site, initially displaying slow movement before undergoing accelerated internalization, indicating that pAPN initiated CME and CavME of TGEV, which also exhibited two distinct stages (Fig 4S and 4X). Moreover, fluorescence images showed that SADS-CoV colocalized with Cla/Cav1 during internalization, but did not colocalize with pAPN (S5A-S5D Fig, S3 Movie). Single-virus tracking also confirmed that SADS-CoV could undergo both CME and CavME independently of pAPN (S5E–S5N Fig), suggesting that pAPN is not essential for SADS-CoV internalization. These results indicate that pAPN can initiate CME and CavME to facilitate PEDV internalization upon binding to PEDV. Although pAPN is not a receptor for PEDV, it initiates PEDV internalization pathways resembling a receptor.

### pAPN initiates PEDV internalization less efficiently than TGEV

**Fig 5:**
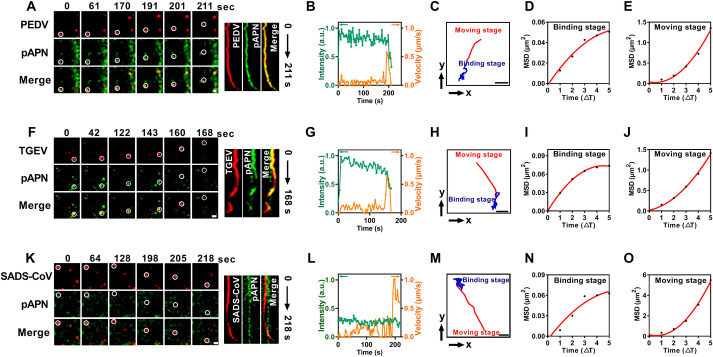
Dynamics of PEDV, TGEV, and SADS-CoV internalization. (A) Time-lapse images and kymographs of PEDV internalization mediated by pAPN. (B) pAPN fluorescence intensities (green line) and velocities (orange line) of the circled PEDV in (A). (C) Trajectories of the circled PEDV in (A), showing the binding stage (blue) and moving stage (red). (D and E) MSD plots of the circled PEDV during the binding stage and the moving stage in (A). (F) Time-lapse images and kymographs of TGEV internalization mediated by pAPN. (G) pAPN fluorescence intensities (green line) and velocities (orange line) of the circled TGEV in (F). (H) Trajectories of the circled TGEV in (F), showing the binding stage (blue) and moving stage (red). (I and J) MSD plots of the circled TGEV during the binding stage and the moving stage in (F). (K) Time-lapse images and kymographs of SADS-CoV internalization. (L) pAPN fluorescence intensities (green line) and velocities (orange line) of the circled SADS-CoV in (K). (M) Trajectories of the circled SADS-CoV in (K), showing the binding stage (blue) and moving stage (red). (N and O) MSD plots of the circled SADS-CoV during the binding stage and the moving stage in (K). Scale bar, 2 µm.

To further explore the dynamics of PEDV internalization mediated by pAPN, we employed single-virus tracking to observe the internalization of PEDV, TGEV, and SADS-CoV in IPECs overexpressing pAPN. In the representative time-lapse images (Fig 5A, S4A Movie), pAPN gradually accumulated around DiD-PEDV at the binding site, consistent with the results observed in the kymographs and fluorescence intensity analysis (Fig 5B). Moreover, the trajectory, velocity, and mean squared displacement (MSD) revealed two stages of PEDV internalization: a binding stage with restricted movement and a moving stage with directional movement (Fig 5C–5E). Similarly, we also observed TGEV internalization events mediated by pAPN (S4B Movie). Time-lapse images and kymographs showed that TGEV could also recruit pAPN to the binding site and undergo rapid internalization after colocalization (Fig 5F), further supported by fluorescence intensity analysis (Fig 5G). Viral movement modes also exhibited that TGEV colocalized with pAPN at a low speed, followed by entry into cells at a rapid speed and directed movement (Fig 5H–5J). Additionally, we visualized the internalization dynamics of PDCoV, which also uses pAPN as a receptor, and the results showed that the internalization dynamics of PDCoV initiated by pAPN were similar to TGEV (S6 Fig, S4C Movie). In contrast, SADS-CoV internalized successfully without recruiting pAPN (Fig 5K and 5L), following the same two stages of binding stage and moving stage (Fig 5M–5O), without pAPN involvement (S4D Movie). Additional time-lapse images and kymographs are shown in S7 Fig and S5 Movie.

**Fig 6:**
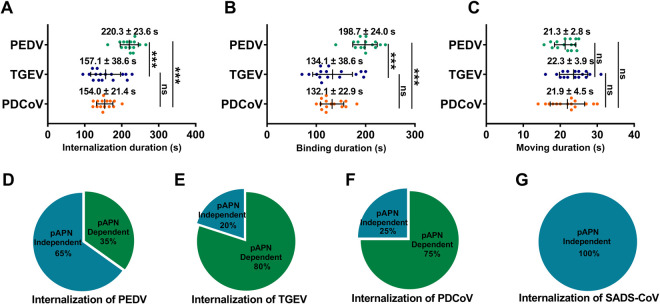
Duration and proportion of internalization events mediated by pAPN. (A) Internalization durations of PEDV (n = 15), TGEV (n = 16) and PDCoV (n = 15) mediated by pAPN. (B) Binding durations of PEDV, TGEV and PDCoV mediated by pAPN. (C) Moving durations of PEDV, TGEV, and PDCoV mediated by pAPN. (D-G) Proportion of pAPN-dependent (PEDV: n = 15; TGEV: n = 16; PDCoV: n = 15; SADS-CoV: n = 0) and pAPN-independent (PEDV: n = 28; TGEV: n = 4; PDCoV: n = 5; SADS-CoV: n = 10) internalization events. Two-tailed *P*-values were calculated by unpaired Student’s t test. *P* < 0.05 was considered significant.

According to these two stages of viral internalization, we analyzed the kinetics of their pAPN-mediated internalization movements. The zero time point of the internalization events is defined as the moment when virions attachment to the cell membrane begins to recruit pAPN. The internalization duration was calculated as the sum of the binding and moving durations. The binding duration was defined as the time elapsed from the beginning of pAPN recruitment to the last frame before the rapid and directed movement of virions. The moving duration was defined as the time elapsed from the beginning of the rapid and directed movement of virions to the last frame before the virions speed slowed. Statistical analysis showed that the internalization durations of PEDV, TGEV, and PDCoV mediated by pAPN were 220.3 ± 23.6 s, 157.1 ± 38.6 s, and 154.0 ± 21.4 s, respectively (Fig 6A). The binding durations of PEDV, TGEV, and PDCoV internalization mediated by pAPN were 198.7 ± 24.0 s, 134.1 ± 38.6 s, and 132.1 ± 22.9 s, respectively (Fig 6B). The moving durations of PEDV, TGEV, and PDCoV internalization mediated by pAPN were 21.3 ± 2.8 s, 22.3 ± 3.9 s, and 21.9 ± 4.5 s, respectively (Fig 6C). Among all internalization events of PEDV, TGEV, PDCoV, and SADS-CoV, pAPN mediated 35% of PEDV internalization events, 80% of TGEV internalization events, and 75% of PDCoV internalization events, but did not participate in SADS-CoV internalization (Fig 6D-6G). These results indicate that the binding duration of pAPN-mediated PEDV internalization is significantly longer than that of TGEV and PDCoV, while the moving duration is similar among the three viruses. Therefore, although pAPN supports PEDV internalization, the process is less efficient in initiating this process compare to its role in TGEV and PDCoV internalization.

To determine the impact of pAPN-facilitated internalization on downstream viral membrane fusion and replication kinetics, we first used DiD-PEDV and DiD-TGEV to infect IPECs overexpressing pAPN at a MOI of 100. When lipophilic dyes label the viral membrane, they spread into a larger area of the cell membrane upon fusion, resulting in increased fluorescence [25]. The fluorescence intensity of a single particle can thus serve as a reporter for membrane fusion. The impact of pAPN-facilitated internalization on PEDV fusion was quantitatively assessed by analyzing the intensity changes of DiD signals from individual viral particles at 30, 60, and 90 mpi. The results showed that DiD-TGEV exhibited significantly higher fluorescence intensity at all three time points compared to DiD-PEDV (S8A and S8B Fig). These findings suggest that the faster internalization of TGEV, mediated by pAPN, results in earlier and more efficient membrane fusion than PEDV. We next investigated whether the increased membrane fusion efficiency resulting from pAPN-facilitated internalization ultimately impacts productive infection. IPECs with or without pAPN transfection were infected at an MOI of 1, and viral titers were measured at multiple time points post-infection. The results showed that pAPN significantly enhanced PEDV titers at 6 and 12 hpi, whereas for TGEV, pAPN enhanced infectious virus production at all measured time points from 6 to 60 hpi (S8C and S8D Fig). For PEDV, pAPN may primarily function as an entry co-factor that facilitates initial internalization and membrane fusion, thereby conferring an early kinetic advantage. However, as the infection progresses, the involvement of other host factors or alternative entry pathways may reduce PEDV’s dependence on pAPN. In contrast, as a functional receptor for TGEV, pAPN is essential for productive infection, and TGEV remains highly reliant on pAPN throughout its entire replication cycle. These results underscore the biological relevance of entry kinetics and support the view that pAPN-mediated internalization not only affects entry efficiency but also shapes the temporal dynamics of infection establishment. The acceleration of early entry steps facilitated by pAPN enables viruses to initiate replication sooner, thereby shortening its overall replication cycle.

## Discussion

In this study, we employed single-virus tracking to quantitatively investigate the exact role of pAPN in PEDV entry at the dynamic level, comparing its function with its established receptor role for TGEV and non-receptor status for SADS-CoV. Our results reveal that pAPN shows a binding affinity for PEDV S protein and facilitates PEDV infection *via* internalization, although it is not a receptor for PEDV. Upon binding, pAPN initiates both CME and CavME pathways to facilitate PEDV internalization, similar to its role in TGEV entry. However, pAPN mediates PEDV internalization less efficiently than it mediates TGEV internalization. Specifically, pAPN is involved in only 35% of PEDV internalization events but in up to 80% of TGEV internalization events. Notably, pAPN-mediated PEDV internalization is approximately 60 s slower than that of TGEV, and we further found that the dynamic differences between pAPN-mediated PEDV and TGEV internalization are mainly due to the binding stage, while the durations of the moving stage are similar. These findings clarify the mechanism and extent of pAPN involvement in the PEDV internalization and highlight the kinetic characteristics of pAPN during PEDV entry.

Receptor refers to a host surface molecule essential for productive viral infection. It binds the virus with high specificity and affinity and initiates critical post-binding events. Its expression can confer susceptibility to otherwise resistant cells, while its deletion abrogates infectivity. In contrast, an attachment factor typically facilitates viral binding without triggering internalization, while an entry co-factor assists internalization but may not independently enable viral infection in otherwise non-permissive cells [26,27]. Previous reports have demonstrated that pAPN indeed promotes PEDV infection in various cell types, with PEDV infection efficiency being correlated with the expression level of pAPN [28–30]. These observations are consistent with our findings, we show that pAPN promotes PEDV infection through an internalization mechanism in susceptible cells. Additionally, molecular docking and SPR analyses demonstrated a specific binding interaction between the PEDV spike protein and pAPN, providing molecular-level evidence for the involvement of pAPN in the PEDV infection. Most notably, single-virus tracking experiments directly visualized the internalization process at the individual virus level and showed that pAPN is involved in approximately 35% of PEDV internalization events. These results provide dynamic and quantitative evidence for the role of pAPN in facilitating PEDV entry. However, several studies have reported that pAPN does not appear to be essential for PEDV infection. The expression of pAPN in HeLa cells failed to render the cells susceptible to PEDV, but they did become susceptible to TGEV [2,12]. Similarly, we also show that pAPN fails to rescue PEDV infection in non-susceptible Caco2 cells. Our findings provide a new perspective that may help reconcile these discrepancies. The role of pAPN in PEDV infection appears to require a permissive cellular background. In non-susceptible cells, such as HeLa and Caco2, pAPN expression alone was insufficient to support productive PEDV infection, suggesting that additional host factors, potentially unidentified functional receptors, might be required. Furthermore, pAPN-knockout pigs remain susceptible to PEDV infection, and PEDV can infect pAPN-negative Vero cells [13,14], further supporting the notion that pAPN is not essential for PEDV infection. Consistently, our single-virus tracking analysis revealed that only a subset (~35%) of PEDV internalization events involve pAPN, indicating that PEDV likely employs multiple entry pathways. Previous studies have demonstrated that multiple host factors—including transferrin receptor 1 (TfR1), sialic acid (SA), and heparan sulfate (HS)—contribute to the entry process of PEDV [31–33]. This mechanistic redundancy could explain why PEDV infection persists in pAPN-knockout pigs or pAPN-negative cells. Therefore, pAPN does not meet the criteria for a functional receptor for PEDV, and our results support the classification of pAPN as an entry co-factor, a molecule that contributes to PEDV internalization in a subset of events and enhances infection efficiency in susceptible cells, but is not essential for viral entry.

**Fig 7:**
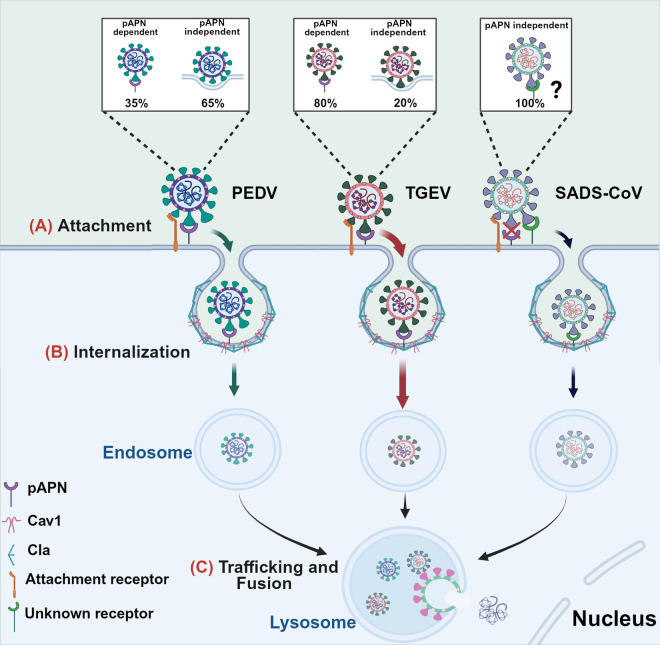
Schematic of PEDV, TGEV and SADS-CoV entry mediated by pAPN. pAPN plays distinct roles in the entry of PEDV, TGEV and SADS-CoV. (A) Initially, PEDV and TGEV but not SADS-CoV, recruit pAPN after adhesion to the cell surface *via* attachment receptors. (B) After binding, pAPN mediates more efficient TGEV internalization compared to PEDV. (C) Finally, PEDV, TGEV and SADS-CoV undergo fusion in the late endosomes/lysosomes after intracellular trafficking. The image was created using the website https://app.biorender.com/.

Surprisingly, despite TGEV recognizing pAPN as a functional receptor with higher affinity than PEDV and SADS-CoV, all three viruses exhibit similar attachment levels in cells overexpressing pAPN. In addition to the direct receptor recognition, attachment receptors, such as HS and SA [34,35], can facilitate the initial contact of CoVs with the cell surface [36]. The presence of attachment receptors collects the virions to the cell surface and reduces the distance between APN and RBD, thereby increasing the probability of APN-RBD interactions [37] (Fig 7A). Importantly, pAPN can initiate PEDV internalization through mechanisms resembling its established receptor-mediated role in TGEV entry. Specifically, single-virus tracking revealed that pAPN-mediated PEDV internalization displays kinetic features similar to those of TGEV, including the activation of both CME and CavME. However, we found that pAPN enhanced the internalization of both PEDV and TGEV, with the promotion of TGEV internalization being more significant than that of PEDV. Consistently, single-virus tracking also showed that pAPN-mediated PEDV internalization is less frequent and slower than that of TGEV and PDCoV. The binding affinity between pAPN and the S proteins remains a key determinant of entry efficiency [38–40]. Our results and previous studies both indicate that pAPN has a higher affinity for TGEV and PDCoV than for PEDV, which may explain why it mediates more efficient internalization of TGEV and PDCoV compared to PEDV. Furthermore, we found that the binding duration of pAPN-mediated PEDV internalization is significantly longer than that of TGEV and PDCoV, which notably restricts the internalization speed of PEDV, while the duration of the moving stage is similar. The binding affinity between pAPN and the viruses tends to regulate internalization dynamics, with higher affinity improving the efficiency of endocytic signaling transduction and reducing internalization time (Fig 7B). Interestingly, we observed that pAPN is involved in 80% of TGEV and 75% of PDCoV internalization events, more than twice the proportion of PEDV internalization events mediated by pAPN. The proportion and duration of pAPN-mediated PDCoV internalization are similar to those of TGEV, further highlighting the similar kinetic characteristics of pAPN as a receptor mediating viral entry. Therefore, dynamic quantitative analysis of host factor-mediated viral entry based on single-virus tracking could serve as a screening tool for receptor identification in the future [41,42].

The distinct binding affinities between pAPN and porcine enteric coronaviruses represent a key molecular basis for the divergent internalization dynamics observed during viral entry. In the case of human coronaviruses, especially SARS-CoV-2, numerous studies have documented that mutations in the spike protein among different variants lead to altered binding affinities for the ACE2 receptor. Variants such as Alpha, Delta, and Omicron exhibit progressively higher affinities for ACE2, which have been associated with increased infectivity, enhanced transmissibility, and distinct entry kinetics [43]. In addition, recent studies have demonstrated that SARS-CoV-2 and other coronaviruses can also utilize various co-factors or attachment factors, including SA, HS, CD147, and TfR1 [44–46]. These auxiliary molecules may act synergistically with the primary receptor to enhance viral entry efficiency or broaden host range by modulating viral internalization dynamics. Here, our study provides a framework for dissecting how subtle molecular differences at the receptor-RBD binding interface modulate binding affinity and, in turn, translate into measurable kinetic changes during viral entry. This approach may facilitate the identification of critical host factors and support the evaluation of viral variants with altered entry properties. Additionally, understanding how receptor binding affinity governs internalization dynamics can inform the rational design of entry inhibitors or decoy receptors that disrupt critical stages of viral entry. Dissecting the kinetic consequences of receptor engagement may aid in predicting the zoonotic potential of emerging coronaviruses based on their ability to engage host receptors with sufficient affinity and internalization efficiency.

While our study clarifies the controversial role of pAPN in PEDV entry and demonstrates that pAPN functions as an entry co-factor facilitating internalization, the functional receptor of PEDV remains unidentified. Future studies aimed at systematically identifying the bona fide PEDV receptor using receptor screening approaches, such as genome-wide CRISPR/Cas9 knockout or overexpression libraries [47], combined with biochemical and kinetic validation, will be essential to fully elucidate the molecular mechanisms underlying PEDV entry.

In conclusion, our study presents a quantitative delineation of pAPN’s specific role in PEDV infection and elucidates the puzzle of pAPN in PEDV infection. These findings not only significantly advance our understanding of the entry mechanisms employed by PEDV, but also provide a theoretical basis for receptor evaluation from a kinetic perspective, thereby offering crucial insights into targeted preventive and therapeutic strategies against PEDV infection.

## Materials and methods

### Cell culture

IPECs, Vero cells, ST cells, LLC-PK1 cells, and Caco2 cells were obtained from the American Type Culture Collection (ATCC). The IPI-2I-APN^-/-^ cell line was kindly provided by Dr. Shaobo Xiao (Huazhong Agricultural University, China) [48]. All cells were cultured in Dulbecco’s modified Eagle’s medium (DMEM) supplemented with high-glucose (Vivacell, China), 10% fetal bovine serum (FBS, Vivacell, China) and 100 U/mL penicillin and streptomycin. All cells used in this study were maintained in a humidified incubator at 37 °C with 5% CO_2_.

### Virus propagation, purification, and labeling

The PEDV CV777 strain (GenBank accession No. AF353511.1), PEDV ZJU strain (GenBank accession No. KU558701.1) and SADS-CoV GDWT strain (GenBank accession No. MG557844) used in this study were propagated in Vero cells. The TGEV H16 strain (GenBank accession No. FJ755618.2) was propagated in ST cells. The PDCoV CZ2020 strain (GenBank accession No. OK546242) was propagated in LLC-PK1 cells. All viruses were treated with trypsin (5 μg/mL, Sigma, USA) at 37 °C for 72 h. After incubation, the viral supernatant was harvested and centrifuged at 12,000 × g for 30 min at 4 °C to remove cell debris. The viruses were then concentrated by ultracentrifugation at 100,000 × g for 2 h at 4 °C. The viruses were further purified using differential centrifugation and 10% - 60% (wt/vol) sucrose gradient ultracentrifugation, and stored at -80 °C. The centrifuge assay was performed using a Beckman SW32Ti rotor (Beckman Coulter, USA). For single-virus tracking, the lipid membranes of viruses were labeled using the lipophilic dye DiD (Thermo Fisher, USA). The purified viruses were incubated with 20 μM DiD dye at room temperature for 2 h with gentle vortex. Excess dye was removed using a NAP-10 filtration column (GE Healthcare, USA). The DiD-labeled viruses were then stored at -80 °C for further use in single-virus tracking experiments.

### Plasmid construction and transfection

mCherry-pAPN and GFP-pAPN were constructed by cloning *pAPN* from IPECs into the pmCherry-C1 and pEGFP-C1 plasmids. GFP-Cla, mCherry-Cla, GFP-Cav1 and mCherry-Cav1 were constructed in our previous study (23). For plasmid transfection, cells were grown in 35-mm petri dishes (Nest Biotechnology, China) until approximately 80% confluence. A transfection mix was prepared by diluting 2 μL of Lipofectamine 3000 reagent (Thermo Fisher, USA) and 1 μg of plasmid DNA in DMEM (Vivacell, China) to a final volume of 100 μL. The mixture was incubated for 20 min at room temperature before being added to the cells. The transfected cells were then incubated at 37 °C with 5% CO_2_ for 24 h.

### Immunofluorescence assay (IFA)

IPECs and IPI-2I-APN^-/-^ cells were infected with viruses at an MOI of 0.1 and incubated at 37 °C for 24 h. After infection, the cells were fixed with 4% paraformaldehyde (Beyotime, China) for 15 min, followed by permeabilization with 0.2% Triton X-100 for 10 min. The cells were then blocked with QuickBlock blocking buffer (Beyotime, China) at room temperature for 15 min. For the detection of viral proteins, the cells were incubated overnight at 4 °C with primary antibodies, including a mouse anti-SADS-CoV N IgG (1:500 dilution, Blue City Biology, China), a mouse anti-PEDV N IgG (1:500 dilution, prepared in our laboratory) or a mouse anti-TGEV N IgG (1:1000 dilution, Blue City Biology, China). The cells were then incubated with a FITC-conjugated goat anti-mouse IgG secondary antibody (1:500 dilution; Biodraon, China) at room temperature for 1 h. Finally, cellular nuclei were stained with Hoechst 33342 (Beyotime, China) and the cells were imaged using a Nikon A1 confocal microscopy (Nikon, Japan).

### Attachment and internalization assays

For attachment assay, IPECs grown to 70% - 90% confluence in 12-well cell culture plates were infected with PEDV, TGEV, or SADS-CoV at an MOI of 10. The cells were incubated with the viruses at 4 °C for 30 min to allow for attachment. After incubation, unbound virions were removed by washing the cells three times with phosphate-buffered saline (PBS). The cells were then lysed using VeZol Reagent (Vazyme Biotech, China) to extract RNA, and the viral RNA levels were quantified by RT-qPCR. For internalization assay, cells were washed three times after attachment, and then cells were incubated in pre-warmed DMEM (Vivacell, China) for 60 min at 37 °C to allow internalization. After incubation, residual plasma membrane-bound virions were removed by treating the cells with proteinase K (0.5 μg/mL) for 120 min at 4 °C. The cells were then washed three more times with PBS, lysed in VeZol Reagent (Vazyme Biotech, China), and the internalized viral RNA levels were also quantified by RT-qPCR.

### Reverse Transcription Quantitative real-time PCR (RT-qPCR)

Total RNA was extracted from cells using the VeZol Reagent (Vazyme Biotech, China) according to the manufacturer’s instruction. First-strand synthesis was performed using a HiScript II 1st Strand cDNA Synthesis Kit (Vazyme Biotech, China). RT-qPCR was conducted on a LightCycler 96 system (Roche LifeScience, Switzerland) using the AceQ qPCR SYBR Green Master Mix (Vazyme Biotech, China). Positive and negative controls were included in each experiment, and all results were normalized to the expression level of *GAPDH*. Quantification of data was performed using the 2^-ΔΔCT^ method. The primers used for RT-qPCR were as follows: TGEV *N* gene primers: 5’-CATGGTGAAGGGCCAACGTA-3’ and 5’-GGCAACCCAGACAACTCCAT-3’, SADS-CoV *N* gene primers: 5’-CTAAAACTAGCCCCACAGGTC-3’ and 5’-TGATTGCGAGAACGAGACTG-3’, PEDV *N* gene primers: 5’-GCACTTATTGGCAGGCTTTGT-3’ and 5’-CCATTGAGAAAAGAAAGTGTCGTAG-3’, and *GAPDH* gene primers: 5’-CCTTCCGTGTCCCTACTGCCAAC-3’ and 5’-GACGCCTGCTTCACCACCTT

CT-3’.

### Western blotting

Cells were washed three times with PBS (pH 7.2) and lysed in RIPA lysis buffer (Vazyme Biotech, China) containing 1 mM protease and phosphatase inhibitors (Beyotime, China). After centrifugation at 13,000 × g for 10 min at 4 °C, the supernatants were collected and the protein content was normalized using a bicinchoninic acid (BCA) protein assay kit (Beyotime, China). Equal amounts of protein were separated by 10% Bis-Tris sodium dodecyl sulfate-polyacrylamide gel electrophoresis (SDS-PAGE), and transferred onto 0.2-μm nitrocellulose blotting membranes (GE Healthcare, USA). The membranes were blocked with 5% nonfat milk for 1 h at room temperature to prevent non-specific binding. After blocking, the membranes were incubated with primary antibodies specific for each target protein overnight at 4 °C. The next day, the membranes were incubated with horseradish peroxidase (HRP)-conjugated secondary antibodies for 1 h at room temperature. The protein bands were then visualized using enhanced chemiluminescence (ECL) buffer (Vazyme Biotech, China) and quantified using Fiji/ImageJ software.

### Single-virus tracking

Cells were initially seeded onto 35-mm petri dishes (Nest Biotechnology, China), and transfected with plasmids for 24 h at 37 °C. The cells were then incubated with DiD-labeled virions (MOI = 100) at 4 °C for 30 min to facilitate virus adsorption. After adsorption, the cells were washed three times with PBS to remove unbound virions. To prepare for live-cell imaging, the culture medium was then replaced with phenol red-free DMEM (Procell Life, China) containing 2% ProLong live antifade reagent (Thermo Fisher, USA), which includes naturally occurring *E. coli* plasma membrane enzymes. For single-virus tracking, the cells were maintained in a stage-top incubator (Tokai Hit, Japan) at 37 °C with 5% CO_2_. Imaging was performed using a commercial Nikon A1 plus confocal microscopy (Nikon, Japan) equipped with an Apo 60 × /1.40 numerical aperture (NA) oil immersion objective lens (Nikon, Japan). The fluorescence signals were captured using an EMCCD camera (Andor, UK) and excited with lasers at wavelengths of 488 nm (Melles Griot, USA), 561 nm (Coherent, USA), and 640 nm (Coherent, USA). Emission filters used included 447/60 nm, 527/55 nm, 615/70 nm, and 707/90 nm (Coherent, USA). Three-dimensional (3D) fluorescence images were obtained using Z-stacks with a step size of 1 μm. More than 200 viral particles were tracked per group across three independent experiments. From these, representative internalization events were selected for quantitative analysis. These events were clearly identified as pAPN-mediated based on defined trajectories, velocity, colocalization with pAPN, and DiD fluorescence signal intensity.

### Phylogenetic analysis and molecular docking

Alignment of multiple sequences and homology analysis of PEDV S, TGEV S and SADS-CoV S were performed using the MegAlign 6.0 (DNASTAR, USA). Amino acid sequences of PEDV, TGEV, and SADS-CoV were visualized based on the online program ESPript algorithm (http://www.ipbs.fr/ESPript) and aligned with the online program CLUSTAL-W (https://www.genome.jp/tools-bin/clustalw). The structure of TGEV S was predicted using I-TASSER (USA), and the structures of PEDV S and SADS-CoV S were downloaded from RCSB PDB database (https://www.rcsb.org/). TGEV S, PEDV S, and SADS-CoV S were docked with pAPN using the online program Cluspro (https://cluspro.org/help.php) and RosettaDock (https://docs.rosettacommons.org). Finally, PyMOL 2.4.0 software (USA) was used for protein dehydration, hydrogenation, and conformational display. The free binding energies obtained from molecular docking were converted to dissociation constants to facilitate comparison with SPR-derived affinities, using the following equation: ΔG = RT ln K_D_.

### SPR assay

The S1 proteins of PEDV, TGEV, and SADS-CoV, each carrying a C-terminal His tag, were expressed and purified using a HEK293T cell expression system. pAPN fused with a hFc tag was also expressed and purified from HEK293T cells. SPR experiments were performed using the Biacore 8K system at room temperature. pAPN was immobilized on the CM5 sensor chips (GE Healthcare, USA) *via* amine coupling. Serially diluted PEDV S1, TGEV S1, and SADS-CoV S1 in HEPES buffer (Genscript, China) were followed over the chips. All the data was processed using the Biacore 8K Evaluation Software version 5.0.

### Data analysis

Time-lapse images, kymographs, and fluorescence intensity were obtained using NIS-Elements AR 4.51.00 (Nikon, Japan) and deconvoluted with a Gaussian filter to remove background noise and enhance image clarity. Fluorescence intensity data was normalized using the equation: x’ = (x-min)/(max-min). Here, x’ represents the normalized data, x is the raw data, and min and max are the minimum and maximum fluorescence intensity values used in the normalization process. The trajectories and velocities were analyzed using Fiji/ImageJ software. MSD was calculated using MATLAB program (MathWorks, USA), and movement modes were determined by binomially fitting MSD using Origin 2022 (USA). Colocalization analysis was evaluated by Pearson correlation coefficient (PCC). Tricolor colocalization was used to assess the extent to which viral particles simultaneously associate with pAPN and Cla/Cav1 during internalization. Specifically, we calculated two ratios: (1) the proportion of viral particles colocalized with both pAPN and Cla/Cav1 relative to those colocalized with Cla/Cav1 alone, and (2) the proportion of viral particles colocalized with both pAPN and Cla/Cav1 relative to those colocalized with pAPN alone. Colocalization analysis was performed using threshold-based segmentation and object-based quantification in Fiji/ImageJ.

### Statistical analysis

Significance was determined using an unpaired Student’s t-test, conducted with Origin 2022 (USA). Data are presented as mean ± standard deviation (SD) and were derived from three independent experiments, unless otherwise indicated. For multiple comparisons, Bonferroni correction was applied, and the reported *P* values are adjusted accordingly. *P*-values < 0.05 were considered as statistically significant, with levels set as ns (no significant difference), *P* ≥ 0.05, *P < 0.05, **P < 0.01, ***P < 0.001, ****P < 0.0001.

## Supporting information

S1 Fig Amino acid sequence alignment of the S proteins from TGEV, PEDV and SADS-CoV. (A) Alignment of the CTD from TGEV, PEDV and SADS-CoV. (B) Homology analysis of the amino acid sequences of the S protein from TGEV, PEDV and SADS-CoV. (C) Summary of interacting amino acids and free binding energies for the docking structures of pAPN with the S proteins of PEDV, TGEV, and SADS-CoV.
(TIF)

S2 Fig Docking structure of pAPN with PDCoV S protein. Docking structure and atomic details illustrating the interaction between PDCoV S (brown) and pAPN (pink).
(TIF)

S3 Fig The role of pAPN in PEDV and TGEV infection in Caco2 cells. (A) Caco2 cells overexpressing GFP-pAPN were infected with PEDV CV777 (0.1 MOI) strain and ZJU (0.1 MOI) strain, and analyzed by western blotting at 24 hpi. (B) Caco2 cells overexpressing GFP-pAPN were infected with TGEV (0.1 MOI), and analyzed by western blotting at 24 hpi.
(TIF)

S4 Fig Verification of DiD labeling efficiency. (A) Fluorescence images of DiD-PEDV (red), DiD-TGEV (red), DiD-SADS-CoV (red) and DiD-PDCoV (red) labeled with anti-N-FITC (green). Scale bar, 10 μm. (B) Colocalization statistics of DiD signals with FITC. Pearson’s correlation coefficient (PCC) was used to calculate the percentage of colocalization. Data were presented as mean values ± SD of three independent experiments.
(TIF)

S5 Fig The internalization of SADS-CoV *via* CME and CavME is independent of pAPN. (A-D) Fluorescence images and triple-color colocalization ratio of SADS-CoV, pAPN and Cla/Cav1 at 60 mpi. Scale bar, 10 µm. (E and F) Time-lapse images and kymographs (DiD-SADS-CoV: red; pAPN: green; Cla/Cav1: white) of SADS-CoV in CME and CavME independent of pAPN. Scale bar, 2 µm. (G) Fluorescence intensities (green and orange lines) and velocities (blue line) of the circled SADS-CoV in (E). (H) Trajectories of the circled SADS-CoV in (E), showing the binding stage (green) and moving stage (orange). Scale bar, 2 µm. (I and J) MSD plots of the circled SADS-CoV during the binding stage and moving stage in (E). (K) Fluorescence intensities (green and orange lines) and velocities (blue line) of the circled SADS-CoV in (F). (L) Trajectories of the circled SADS-CoV in (F), showing the binding stage (green) and moving stage (orange). Scale bar, 2 µm. (M and N) MSD plots of the circled SADS-CoV during the binding stage and moving stage in (F).
(TIF)

S6 Fig Dynamics of PDCoV internalization. (A and B) Time-lapse images and kymographs of PDCoV internalization mediated by pAPN. (C) pAPN fluorescence intensities (green line) and velocities (orange line) of the circled PDCoV in (A). (D) Trajectories of the circled PDCoV in (A), showing the binding stage (blue) and moving stage (red). (E and F) MSD plots of the circled PDCoV during the binding stage and the moving stage in (A). Scale bar, 2 µm.
(TIF)

S7 Fig Dynamics of PEDV, TGEV, PDCoV and SADS-CoV internalization. (A) Time-lapse images and kymographs of PEDV internalization mediated by pAPN. (B) pAPN fluorescence intensities (green line) and velocities (orange line) of the circled PEDV in (A). (C) Trajectories of the circled PEDV in (A), showing the binding stage (blue) and moving stage (red). (D and E) MSD plots of the circled PEDV during the binding stage and the moving stage in (A). (F) Time-lapse images and kymographs of TGEV internalization mediated by pAPN. (G) pAPN fluorescence intensities (green line) and velocities (orange line) of the circled TGEV in (F). (H) Trajectories of the circled TGEV in (F), showing the binding stage (blue) and moving stage (red). (I and J) MSD plots of the circled TGEV during the binding stage and the moving stage in (F). (K) Time-lapse images and kymographs of PDCoV internalization mediated by pAPN. (L) pAPN fluorescence intensities (green line) and velocities (orange line) of the circled PDCoV in (K). (M) Trajectories of the circled PDCoV in (K), showing the binding stage (blue) and moving stage (red). (N and O) MSD plots of the circled PDCoV during the binding stage and the moving stage in (K). (P) Time-lapse images and kymographs of SADS-CoV internalization. (Q) pAPN fluorescence intensities (green line) and velocities (orange line) of the circled SADS-CoV in (P). (R) Trajectories of the circled SADS-CoV in (P), showing the binding stage (blue) and moving stage (red). (S and T) MSD plots of the circled SADS-CoV during the binding stage and the moving stage in (P). Scale bar, 2 µm.
(TIF)

S8 Fig The effect of pAPN on membrane fusion and infectious titers of PEDV and TGEV. (A) Fluorescence images of IPECs infected with DiD-PEDV and DiD-TGEV at 30, 60, and 90 mpi. Scale bar, 10 µm. (B) Fluorescence intensity of individual virions from (A) (three experiments; mean ± SD). (C and D) Growth curves of PEDV and TGEV measured by TCID_50_ assay. Two-tailed *P*-values were calculated by unpaired Student’s t test. *P < *0.05 was considered significant (ns *P *≥ 0.05, *P < 0.05, **P < 0.01, ***P < 0.001, ****P < 0.0001).
(TIF)

S9 Fig Full western blot analyses. (PDF)

S1 Movie CME and CavME of PEDV initiated by pAPN. (A) CME of PEDV initiated by pAPN, corresponding to the time-lapse images in Fig 4E. (B) CavME of PEDV initiated by pAPN, corresponding to the time-lapse images in Fig 4F.
(MP4)

S2 Movie CME and CavME of TGEV initiated by pAPN. (A) CME of TGEV initiated by pAPN, corresponding to the time-lapse images in Fig 4Q. (B) CavME of TGEV initiated by pAPN, corresponding to the time-lapse images in Fig 4R.
(MP4)

S3 Movie CME and CavME of SADS-CoV independent of pAPN. (A) CME of SADS-CoV independent of pAPN, corresponding to the time-lapse images in S5E Fig. (B) CavME of SADS-CoV independent of pAPN, corresponding to the time-lapse images in S5F Fig.
(MP4)

S4 Movie Dynamic of PEDV, TGEV, PDCoV, and SADS-CoV internalization mediated by pAPN. (A) PEDV internalization mediated by pAPN, corresponding to the time-lapse images in Fig 5A. (B) TGEV internalization mediated by pAPN, corresponding to the time-lapse images in Fig 5F. (C) PDCoV internalization mediated by pAPN, corresponding to the time-lapse images in S6 Fig. (D) SADS-CoV internalization independent of pAPN, corresponding to the time-lapse images in Fig 5K.
(MP4)

S5 Movie Additional internalization events of PEDV, TGEV, PDCoV, and SADS-CoV mediated by pAPN. (A) PEDV internalization mediated by pAPN, corresponding to the time-lapse images in S7A Fig. (B) TGEV internalization mediated by pAPN, corresponding to the time-lapse images in S7F Fig. (C) PDCoV internalization mediated by pAPN, corresponding to the time-lapse images in S7K Fig. (D) SADS-CoV internalization independent of pAPN, corresponding to the time-lapse images in S7P Fig.
(MP4)

S1 Data Source data files. Excel files with separate sheets containing the numerical data for Figs 1–6 and S4–S8.
(XLSX)
