# Supplementary figures and images for: Resolving the APN controversy in PEDV infection: Comparative kinetic characterization through single-virus tracking
Source: PLoS Pathog. 2025 Jun 30;21(6):e1013317. doi: 10.1371/journal.ppat.1013317 (PMC12233901; doi:10.1371/journal.ppat.1013317)

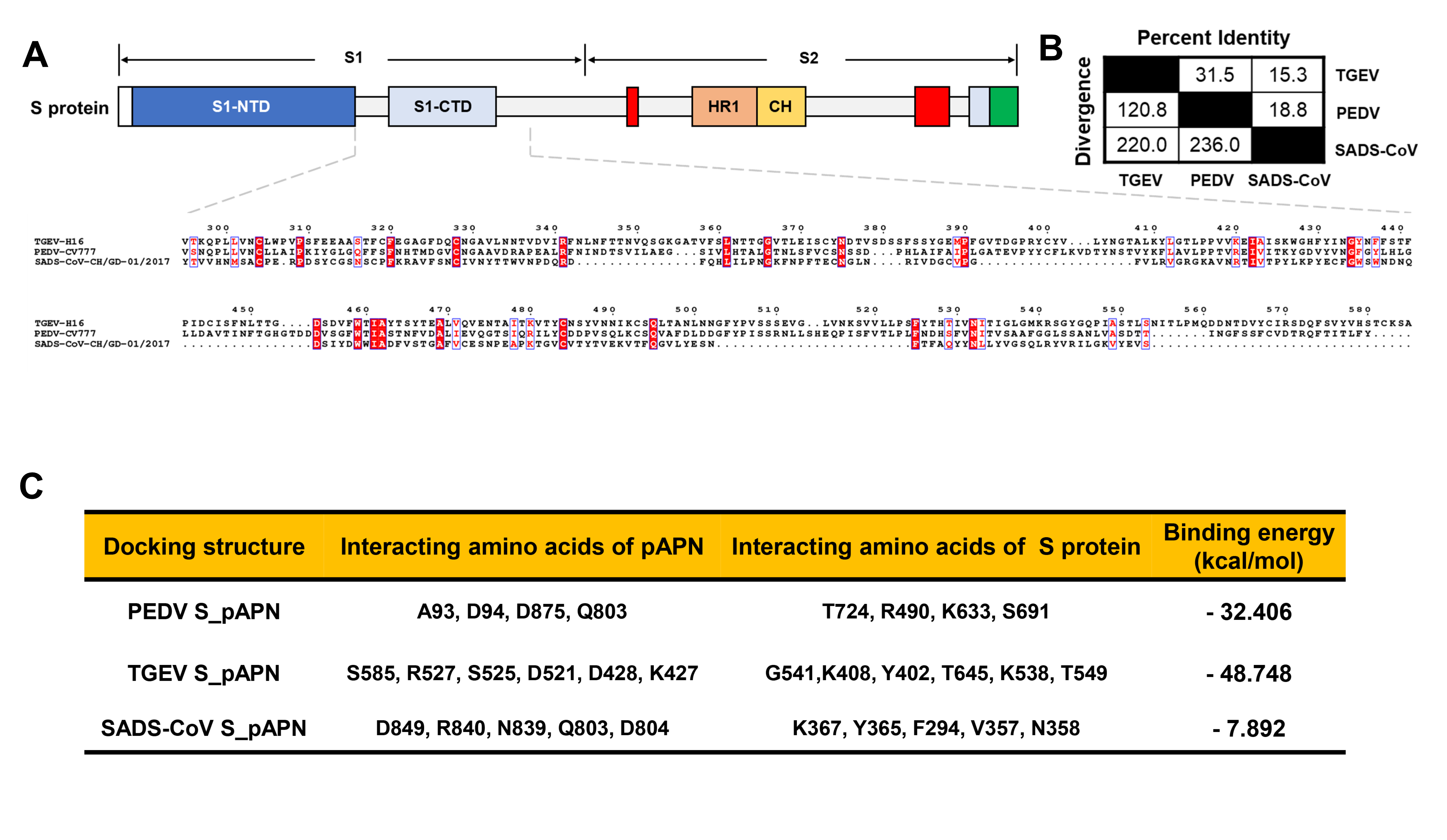

Supplement: S1 Fig — (A) Alignment of the CTD from TGEV, PEDV and SADS-CoV. (B) Homology analysis of the amino acid sequences of the S protein from TGEV, PEDV and SADS-CoV. (C) Summary of interacting amino acids and free binding energies for the docking structures of pAPN with the S proteins of PEDV, TGEV, and SADS-CoV. (TIF) [file ppat.1013317.s001.tif]

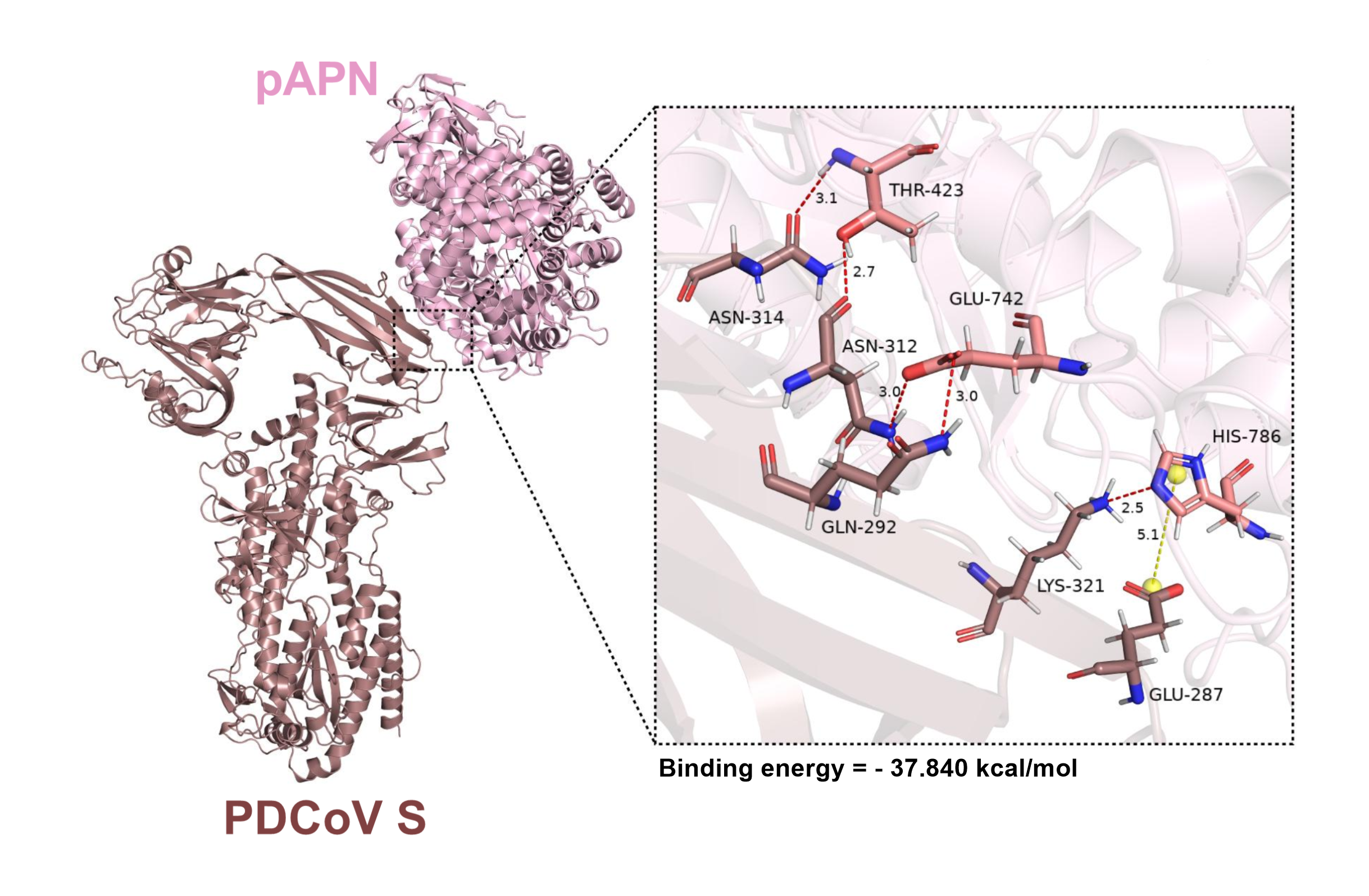

Supplement: S2 Fig — Docking structure and atomic details illustrating the interaction between PDCoV S (brown) and pAPN (pink). (TIF) [file ppat.1013317.s002.tif]

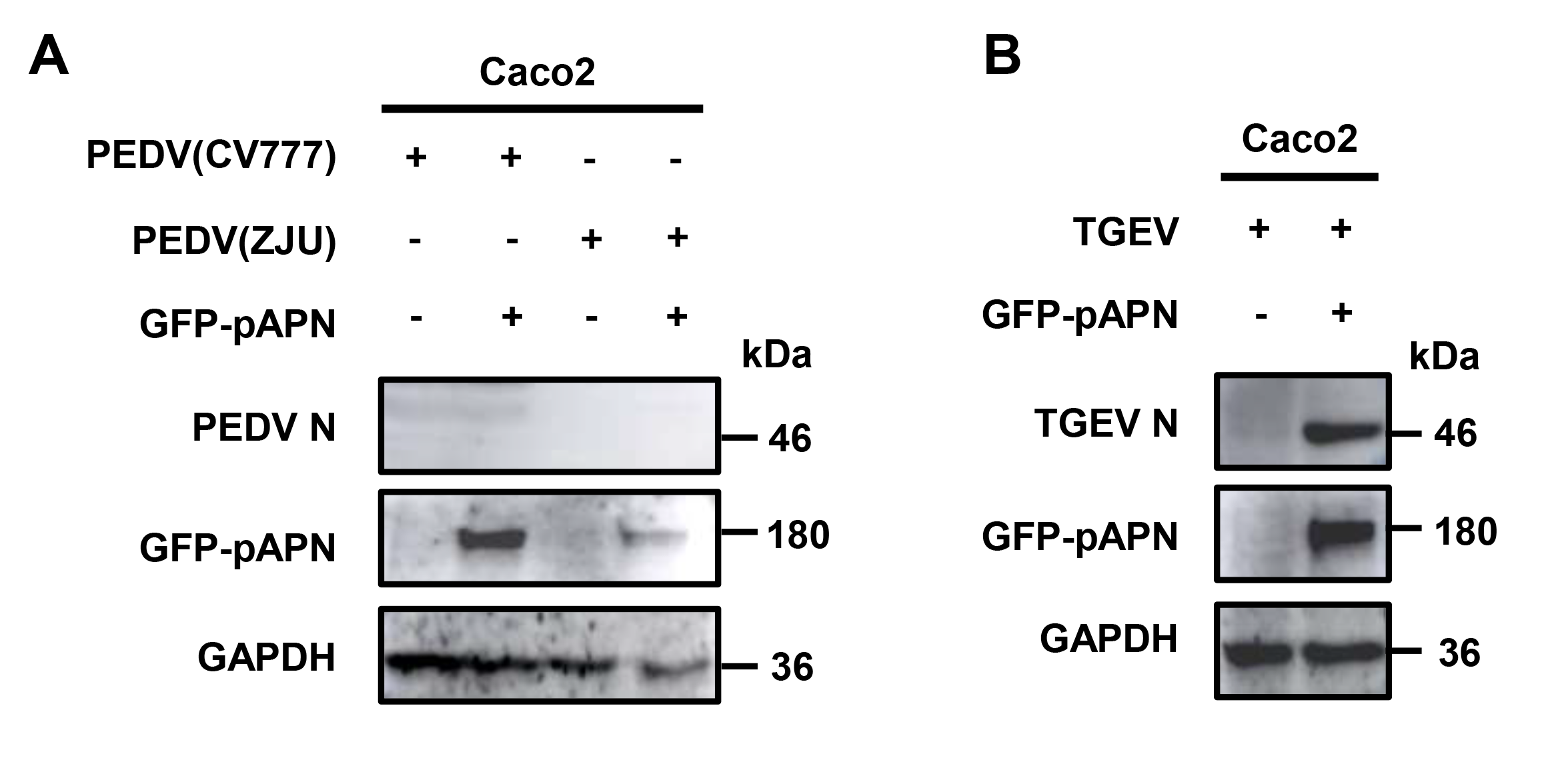

Supplement: S3 Fig — (A) Caco2 cells overexpressing GFP-pAPN were infected with PEDV CV777 (0.1 MOI) strain and ZJU (0.1 MOI) strain, and analyzed by western blotting at 24 hpi. (B) Caco2 cells overexpressing GFP-pAPN were infected with TGEV (0.1 MOI), and analyzed by western blotting at 24 hpi. (TIF) [file ppat.1013317.s003.tif]

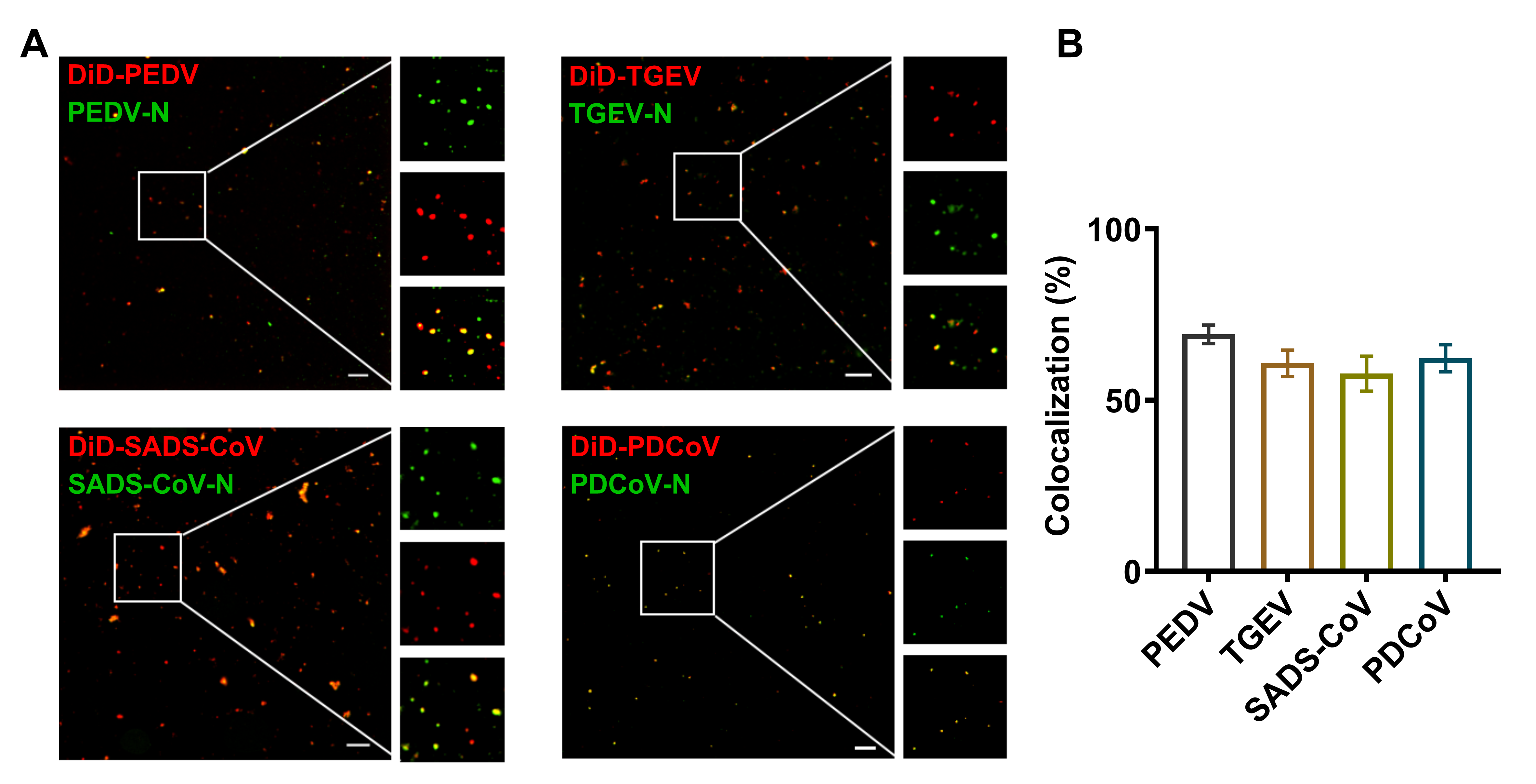

Supplement: S4 Fig — (A) Fluorescence images of DiD-PEDV (red), DiD-TGEV (red), DiD-SADS-CoV (red) and DiD-PDCoV (red) labeled with anti-N-FITC (green). Scale bar, 10 μm. (B) Colocalization statistics of DiD signals with FITC. Pearson’s correlation coefficient (PCC) was used to calculate the percentage of colocalization. Data were presented as mean values ± SD of three independent experiments. (TIF) [file ppat.1013317.s004.tif]

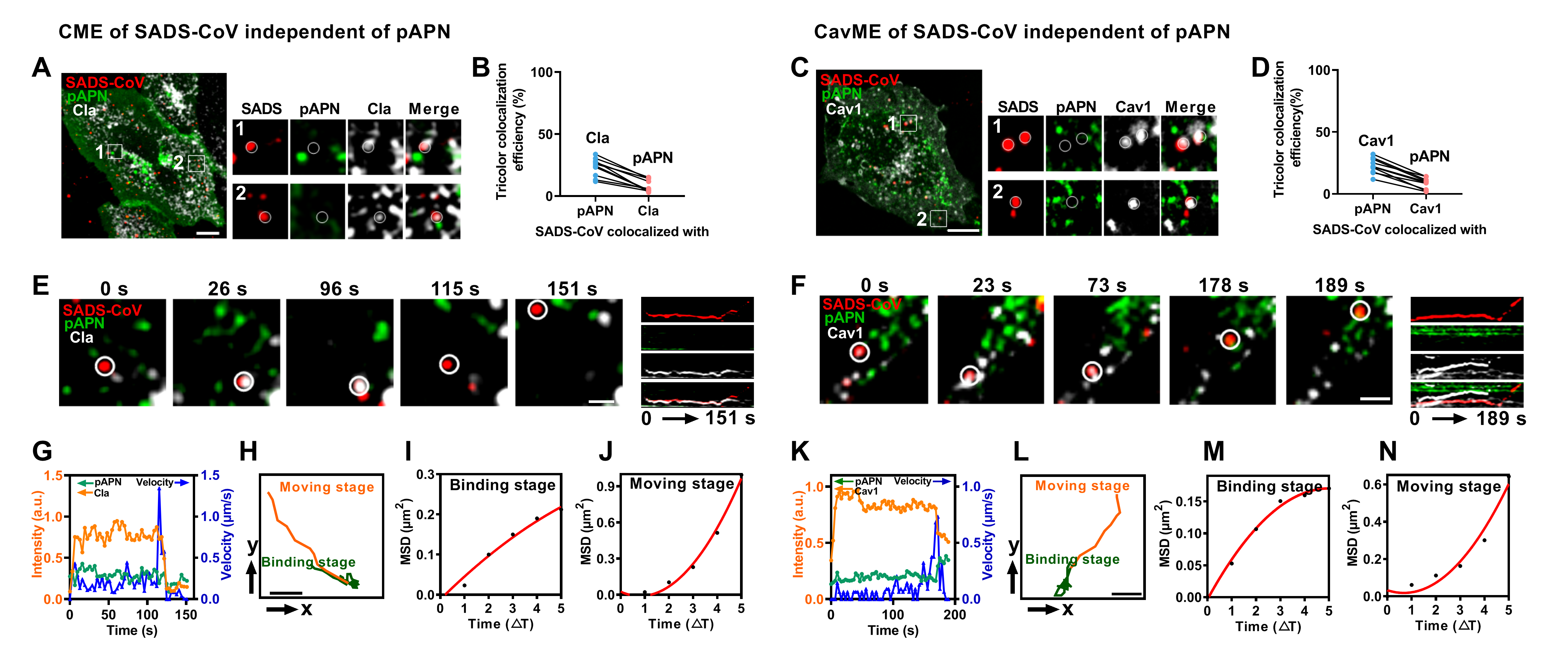

Supplement: S5 Fig — (A-D) Fluorescence images and triple-color colocalization ratio of SADS-CoV, pAPN and Cla/Cav1 at 60 mpi. Scale bar, 10 µm. (E and F) Time-lapse images and kymographs (DiD-SADS-CoV: red; pAPN: green; Cla/Cav1: white) of SADS-CoV in CME and CavME independent of pAPN. Scale bar, 2 µm. (G) Fluorescence intensities (green and orange lines) and velocities (blue line) of the circled SADS-CoV in (E). (H) Trajectories of the circled SADS-CoV in (E), showing the binding stage (green) and moving stage (orange). Scale bar, 2 µm. (I and J) MSD plots of the circled SADS-CoV during the binding stage and moving stage in (E). (K) Fluorescence intensities (green and orange lines) and velocities (blue line) of the circled SADS-CoV in (F). (L) Trajectories of the circled SADS-CoV in (F), showing the binding stage (green) and moving stage (orange). Scale bar, 2 µm. (M and N) MSD plots of the circled SADS-CoV during the binding stage and moving stage in (F). (TIF) [file ppat.1013317.s005.tif]

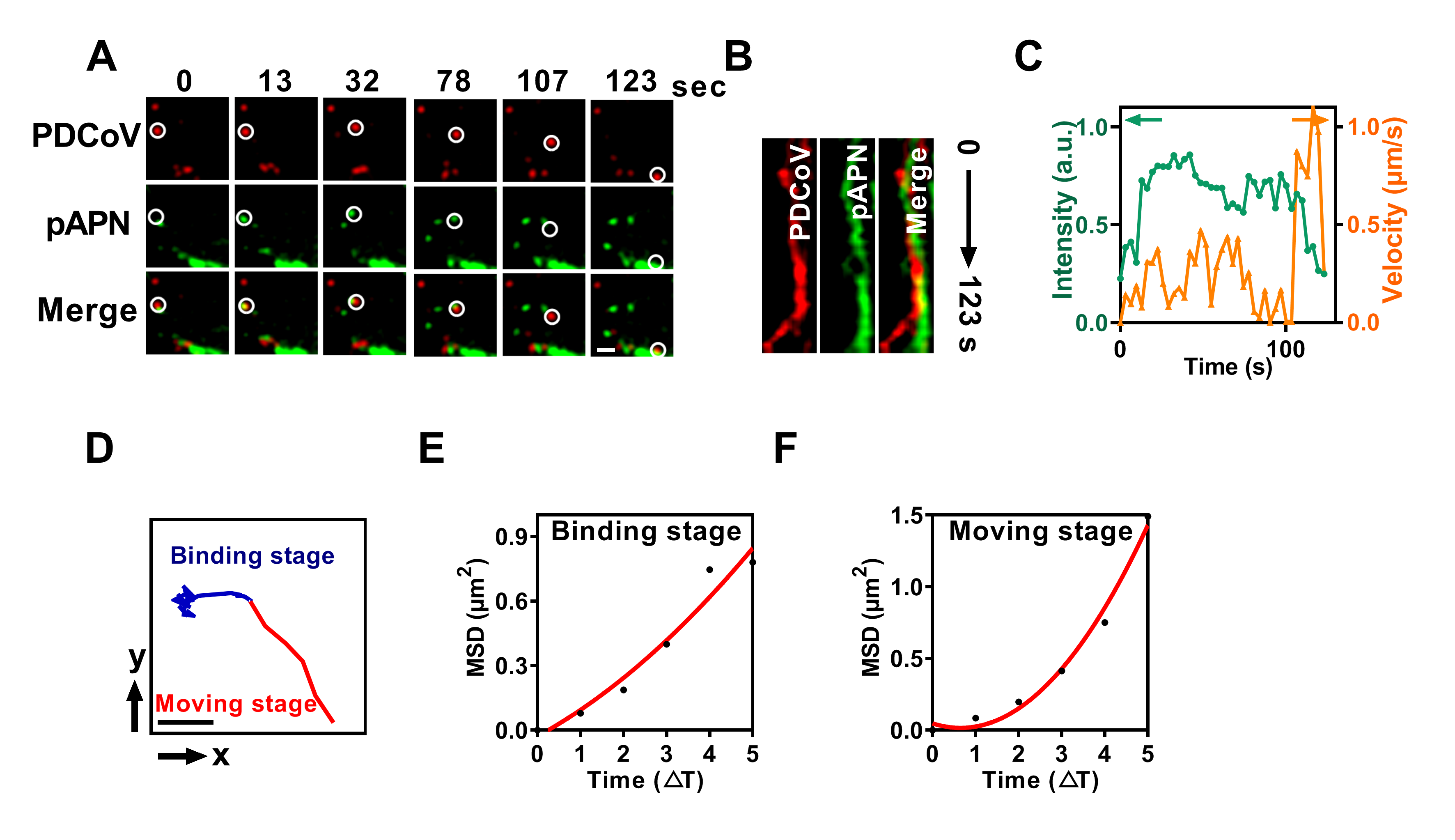

Supplement: S6 Fig — (A and B) Time-lapse images and kymographs of PDCoV internalization mediated by pAPN. (C) pAPN fluorescence intensities (green line) and velocities (orange line) of the circled PDCoV in (A). (D) Trajectories of the circled PDCoV in (A), showing the binding stage (blue) and moving stage (red). (E and F) MSD plots of the circled PDCoV during the binding stage and the moving stage in (A). Scale bar, 2 µm. (TIF) [file ppat.1013317.s006.tif]

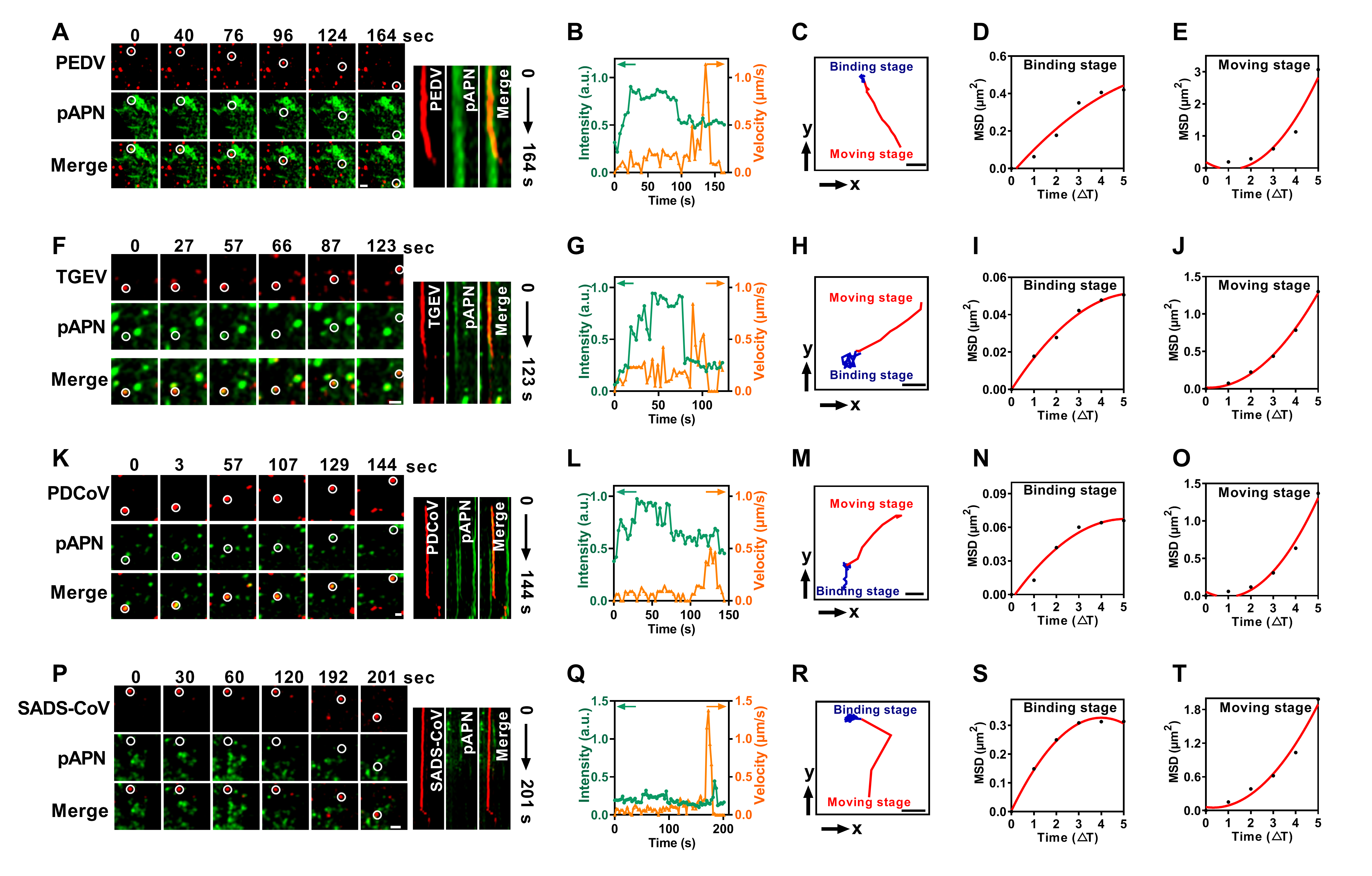

Supplement: S7 Fig — (A) Time-lapse images and kymographs of PEDV internalization mediated by pAPN. (B) pAPN fluorescence intensities (green line) and velocities (orange line) of the circled PEDV in (A). (C) Trajectories of the circled PEDV in (A), showing the binding stage (blue) and moving stage (red). (D and E) MSD plots of the circled PEDV during the binding stage and the moving stage in (A). (F) Time-lapse images and kymographs of TGEV internalization mediated by pAPN. (G) pAPN fluorescence intensities (green line) and velocities (orange line) of the circled TGEV in (F). (H) Trajectories of the circled TGEV in (F), showing the binding stage (blue) and moving stage (red). (I and J) MSD plots of the circled TGEV during the binding stage and the moving stage in (F). (K) Time-lapse images and kymographs of PDCoV internalization mediated by pAPN. (L) pAPN fluorescence intensities (green line) and velocities (orange line) of the circled PDCoV in (K). (M) Trajectories of the circled PDCoV in (K), showing the binding stage (blue) and moving stage (red). (N and O) MSD plots of the circled PDCoV during the binding stage and the moving stage in (K). (P) Time-lapse images and kymographs of SADS-CoV internalization. (Q) pAPN fluorescence intensities (green line) and velocities (orange line) of the circled SADS-CoV in (P). (R) Trajectories of the circled SADS-CoV in (P), showing the binding stage (blue) and moving stage (red). (S and T) MSD plots of the circled SADS-CoV during the binding stage and the moving stage in (P). Scale bar, 2 µm. (TIF) [file ppat.1013317.s007.tif]

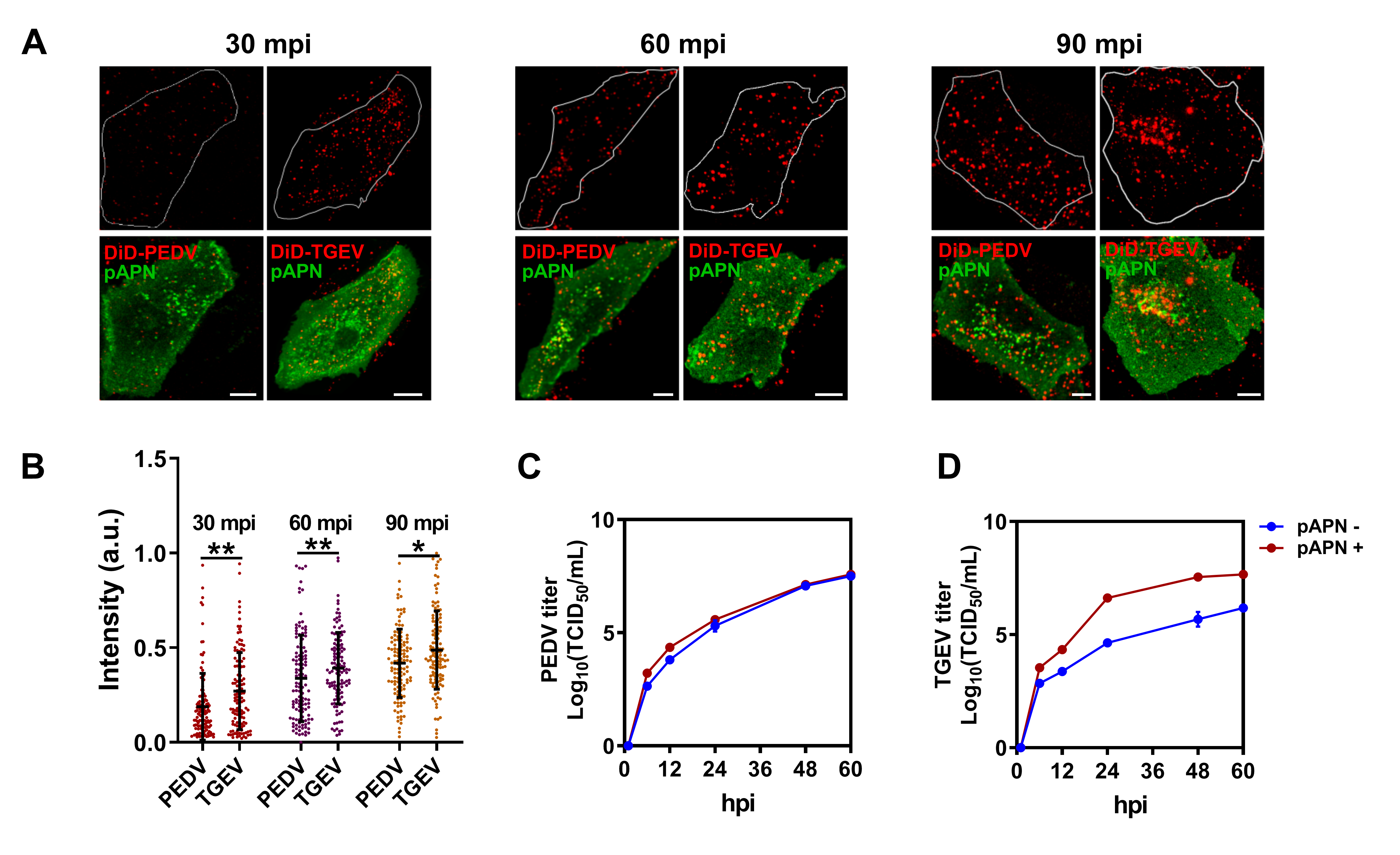

Supplement: S8 Fig — (A) Fluorescence images of IPECs infected with DiD-PEDV and DiD-TGEV at 30, 60, and 90 mpi. Scale bar, 10 µm. (B) Fluorescence intensity of individual virions from (A) (three experiments; mean ± SD). (C and D) Growth curves of PEDV and TGEV measured by TCID50 assay. Two-tailed P-values were calculated by unpaired Student’s t test. P < 0.05 was considered significant (ns P ≥ 0.05, *P < 0.05, **P < 0.01, ***P < 0.001, ****P < 0.0001). (TIF) [file ppat.1013317.s008.tif]

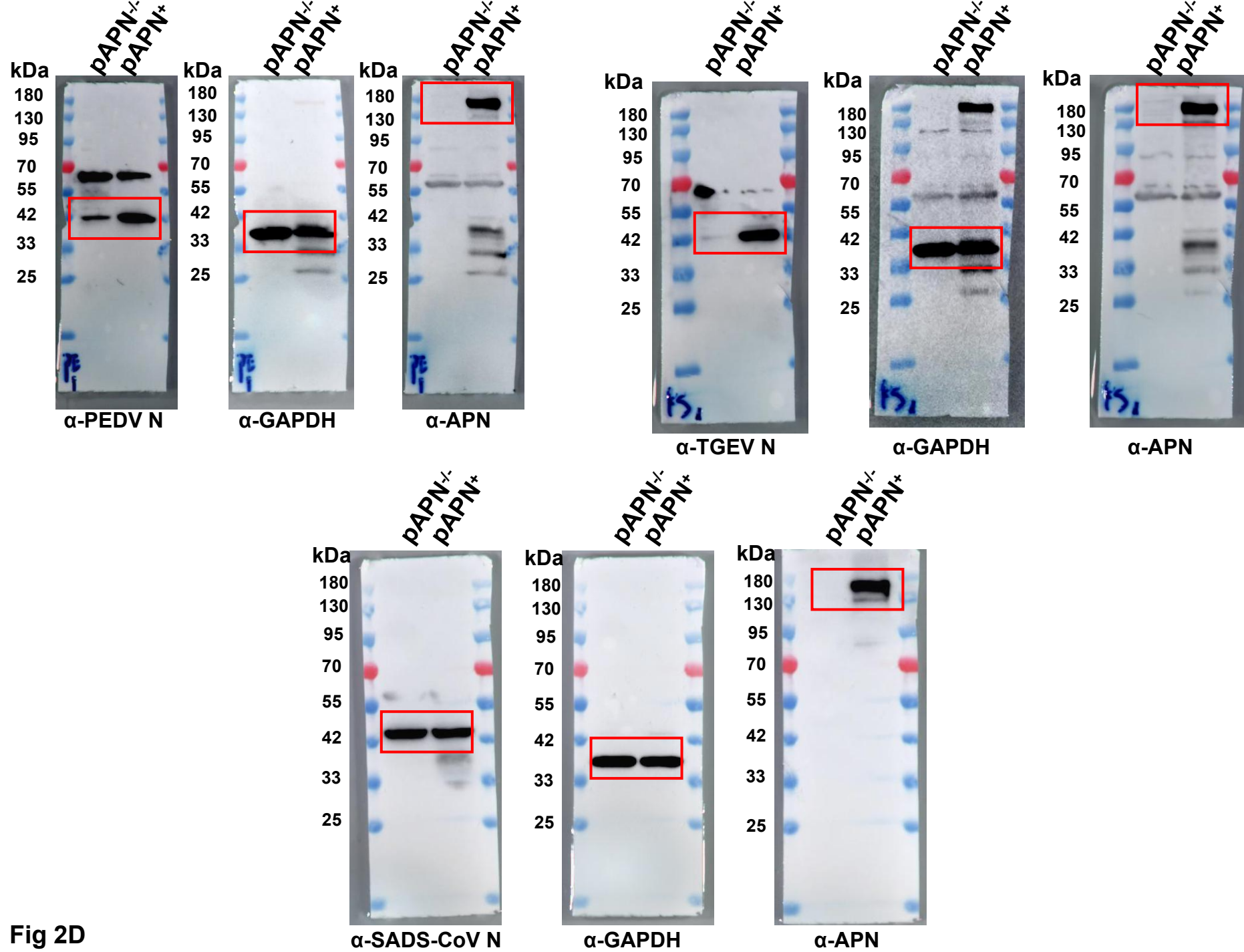

Fig 2D

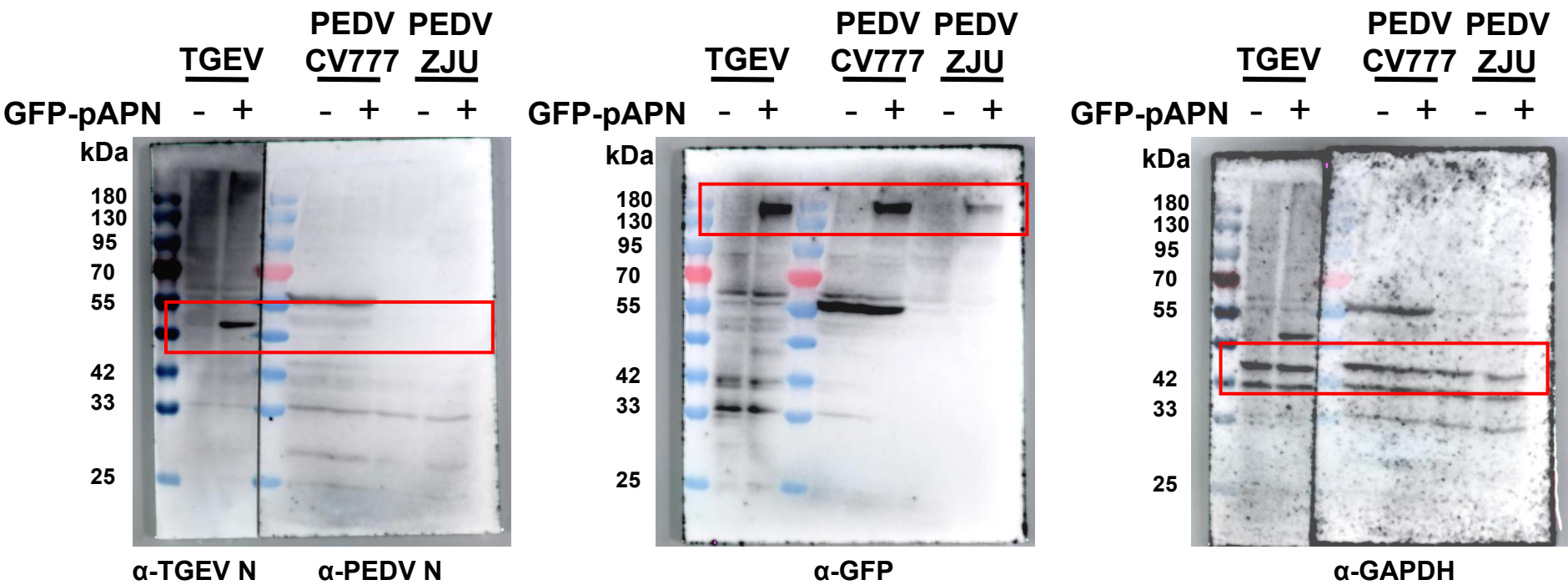

Fig S3

Supplement: S9 Fig — (PDF) [file ppat.1013317.s009.pdf]
